# Supplementary material for: Recycling fossil infrastructure for cleaner energy transitions
Source: Nat Commun. 2026 Mar 15;17:4003. doi: 10.1038/s41467-026-70777-6 (PMC13136374; doi:10.1038/s41467-026-70777-6)
Supplement: Supplementary file 1 — Supplementary Information [file 41467_2026_70777_MOESM1_ESM.pdf]

Supplementary information to "Recycling fossil infrastructure for cleaner energy transitions"

Hauke Schlesier[1,2], Gonzalo Guillén-Gosálbez*[2], Harald Desing*[1]

[1] Technology and Society Laboratory, Empa – Swiss Federal Laboratories for Materials Science and Technology, St. Gallen, Lerchenfeldstrasse 5, 9014, Switzerland.
[2] Institute for Chemical and Bioengineering, Department of Chemistry and Applied Biosciences, ETH Zürich, Vladimir-Prelog-Weg 1, 8093 Zürich, Switzerland.
* corresponding author:
`gonzalo.guillen.gosalbez@chem.ethz.ch`
`harald.desing@empa.ch`

Contents

Supplementary Methods **2**
Supplementary Methods 1: Steel and copper stocks by infrastructure type 2
Supplementary Methods 2: Recycling capacity projections 3
Supplementary Methods 3: Mitigation pathways and temperature trajectories of scenarios 5
Supplementary Methods 4: Life cycle assessment 6
Supplementary Methods 5: Steel and copper production cost 17
Supplementary Methods 6: Steel use in photovoltaic systems 19
Supplementary Methods 7: Material intensities of fossil infrastructure and their uncertainty 21
Supplementary Methods 8: Life cycle impact indicators 23

Supplementary Discussion **25**
Limitations and robustness of results 25
Carbon footprints of green hydrogen and methanol 27
Value of scrap in fossil infrastructure 29

Supplementary Tables **30**
Supplementary Table 7: Global annual production of materials 30
Supplementary Table 8: Steel and copper intensities of clean energy infrastructure 30

Supplementary Figures **31**
Supplementary Figure 18: Total avoided environmental impacts through steel recycling (optimistic) 31
Supplementary Figure 19: Total avoided environmental impacts through steel recycling (pessimistic) 32
Supplementary Figure 20: Process contributions to the environmental impacts of reinforcing steel 33
Supplementary Figure 21: Process contributions to the environmental impacts of low-alloyed steel 34
Supplementary Figure 22: Process contributions to the environmental impacts of chromium steel . 35
Supplementary Figure 23: Total avoided environmental impacts through copper recycling (optimistic) 36
Supplementary Figure 24: Total avoided environmental impacts through copper recycling (pessimistic) 37
Supplementary Figure 25: Process contributions to the environmental impacts of copper 38
Supplementary Figure 26: Externality costs of producing primary or secondary steel 39
Supplementary Figure 27: Externality costs of producing primary or secondary copper 40
Supplementary Figure 28: Environmental benefits of constructing hydrogen electrolyzers and electricity transmission infrastructure with recycled steel and copper 41

Supplementary References **42**

Supplementary Methods

Supplementary Methods 1: Steel and copper stocks by infrastructure type

Supplementary Figures 1a and b show steel and copper stock shares by fossil infrastructure type. Most steel is contained in oil and gas extraction equipment (46%), followed by power plants (26%), pipelines (21%), and coal mines (6%). Regarding copper, 89% of it is contained in power plants, followed by 11% in oil pipelines. The copper in fossil infrastructure is exclusively high-purity (>99.997% purity), and is mostly used in wires and cables. The most common steel type in fossil infrastructure is reinforcing steel (95%) for structural purposes, followed by low-alloyed steel (4%), and chromium steel (stainless steel, 2%; see Supplementary Fig. 1c).

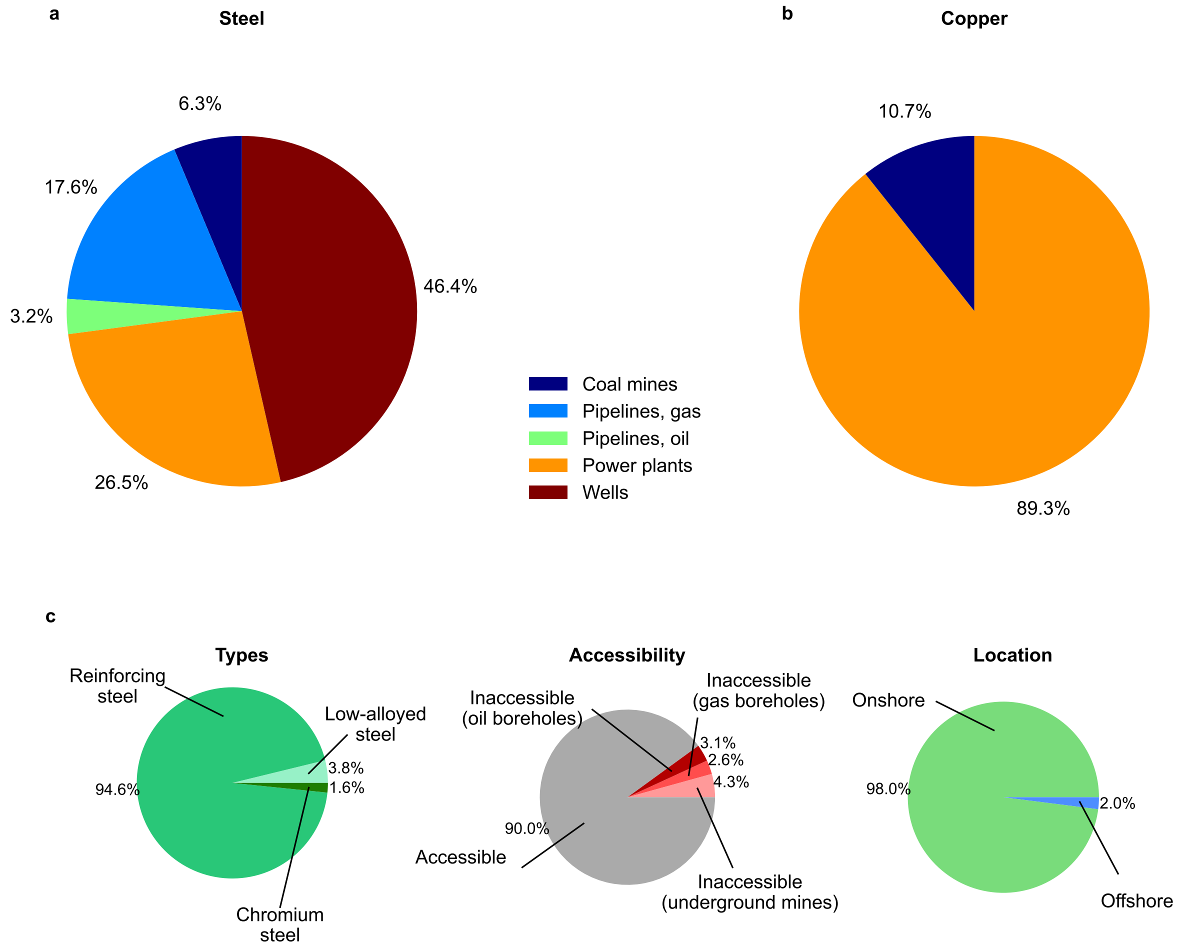

Supplementary Figure 1: **Steel and copper stock percentage by fossil infrastructure type. a**, Share of steel stocks by fossil infrastructure type. **b**, Share of copper stocks by fossil infrastructure type. **c**, Share of steel stocks by steel types, accessibility, and location.

Supplementary Methods 2: Recycling capacity projections

To assess the feasibility of recycling, we estimate the idle recycling capacities (Fig. 3 in the main manuscript) for steel and copper for the period 2025 to 2050. We determine the steel idle recycling capacity from the secondary steel production taken from the outputs of the Integrated Assessment Model scenarios (data source ref.[1]) and capacity utilization factors (see Supplementary Fig. 2). We suspect the secondary steel production projected in the SSP1-NDC (Shared Socioeconomic Pathway 1 Nationally Determined Contributions) scenario for the year 2025 to be an error (84 Mt a^{-1} or only 16% of the current secondary steel production), and instead assume it to be the same as the in SSP1-NPi (National Policies implemented) scenario for the year 2025 (the secondary steel production in 2025 varies little between other scenarios).

We extrapolate the secondary copper production to 2050 using linear regression (R^2=0.90) of historical secondary copper production between 2005 and 2024 (data source ref.[2]), yielding an increase of 172 kt a^{-1} secondary copper production. We then estimate the idle recycling capacity that could be used for handling additional waste streams from the secondary production and the capacity utilization factor as in Supplementary Equation (1) and (2):

$$C_{recycling} = \frac{P_{secondary}}{f_{utilization}} \tag{1}$$

$$C_{recycling,idle} = C_{recycling} \cdot (1 - f_{utilization}) \tag{2}$$

with $C_{recycling}$ being the recycling capacity, $P_{secondary}$ being the secondary production, $C_{recycling,idle}$ being the idle recycling capacity, and $f_{utilization}$ being the capacity utilization factor. For steel, we apply the recycling capacity utilization factor of 0.73 (743 Mt a^{-1} capacity with 541 Mt a^{-1} annual production, see Results section in the main manuscript) with a sensitivity range of 10% (0.66–0.80) for possible future deviation from the current value. For copper, we apply the current secondary copper refinery utilization factor of 0.83[2] (±10%, 0.75–0.91). The recycling capacity for steel and copper are depicted in Supplementary Figure 2a and b.

We then assess the share of idle recycling capacity (Supplementary Eq. 2) taken up for handling the annual volume of scrap released, assuming an even release between 2025 and 2050 (see Supplementary Fig. 2c and d). For steel, the share of idle recycling capacity never exceeds 74% in any year or scenario. For copper, the share of idle recycling capacity never exceeds 30%.

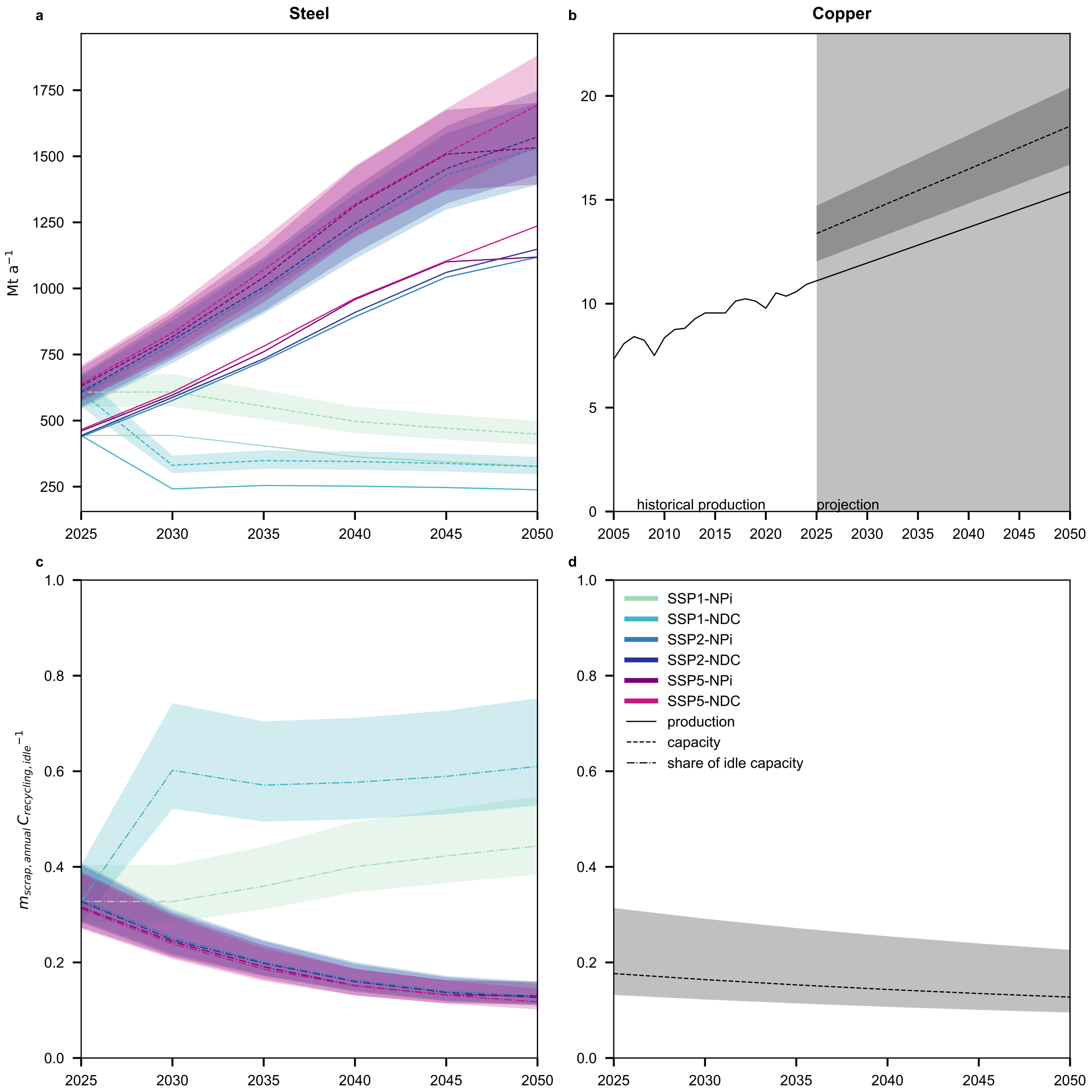

Supplementary Figure 2: **Steel and copper secondary capacity projections. a**, Secondary steel production and production capacity projections for different scenarios (production data from ref.[1], capacity calculated using Supplementary Eq.1). Shaded areas indicate sensitivity to $f_{utilization}$ (±10%). SSP=Shared socio-economic pathway, NPi= National policies implemented, NDC=National determined contributions, Mt=megatons, a=year. **b**, Secondary copper production projection from linear regression between 2005 and 2024 (data source ref.[2]) Dark gray shaded area indicates sensitivity to $f_{utilization}$ (±10%). Production capacity calculated using Supplementary Eq.1. **c, d**, Share of idle capacity ($C_{recyling,\,idle}$, from Supplementary Eq. 2) utilized by annual (**c**) steel and (**d**) copper scrap volume ($m_{scrap,\,annual}$), assuming even release from 2025–2050 and constant $f_{utilization}$ over time. The shaded areas show sensitivity to capacity utilization (±10%).

Supplementary Methods 3: Mitigation pathways and temperature trajectories of scenarios

In this study, we investigate three SSPs—SSP1 (Sustainability), SSP2 (Middle-of-the-road), and SSP5 (Fossil-fueled development)—, which are the SSPs available in Premise v2.2.3.[3] We further investigate two variations: NDC, which are domestic climate mitigation efforts each party to the Paris Agreement has to prepare and communicate in order to fulfill the collective target,[4] and NPi, which are climate policies currently implemented.

Supplementary Figure 3a shows the climate mitigation pathways according to investigated scenarios. Transition times are 70 years or longer and even more than 25 years in very ambitious scenarios (SSP1-PkBudg900; cumulative carbon budget does not exceed 900 Gt CO_2). The time required to recycle steel in current fossil infrastructure (five to 14 years with continental steel waste trade) with idle capacity is therefore well within time periods of typical transitions. Recycling can thus contribute to steel supply in the transition.

Supplementary Figure 3b shows global mean temperature trajectories. Scenarios such as SSP1-NPi, SSP2-NDC, and SSP1-NPi lead to global warming similar to what is expected today (2.5 to 2.9 °C in 2100;[5] see Supplementary Fig. 3b).

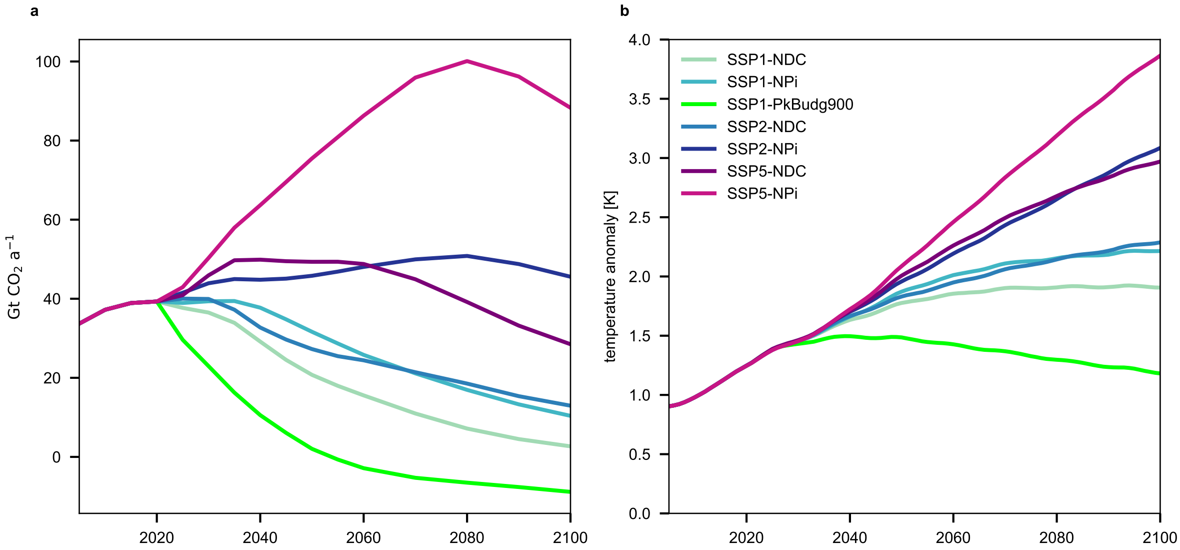

Supplementary Figure 3: **Carbon dioxide mitigation pathways and temperature trajectories. a**, Carbon dioxide mitigation pathways of investigated scenarios and SSP1-PkBudg900. All investigated pathways have a transition time of multiple decades, even very ambitious scenarios (SSP1-PkBudg900) have a transition time of around 30 years. SSP=Shared socio-economic pathway, NPi= National policies implemented, NDC=National determined contributions, Gt=gigatons, a=year, PkBudg900=peak budget 900 Gt CO_2. **b**, Projected temperature increases compared to pre-industrial scenarios until 2100 are shown in the right panel. End-of-century temperature increases of investigated scenarios range from 1.9 °C to 3.9 °C. Data source ref.[73]

Supplementary Methods 4: Life cycle assessment

Goal and scope for steel life cycle assessment

The functional unit of the life cycle assessment is the production of the steel equivalent to the amount currently stored in fossil infrastructure—either by the secondary (electric arc furnace) or primary route (blast furnace and basic oxygen furnace). The geographical scope is global and the temporal scope is 2025 to 2050 in five-year intervals.

System boundaries for the life cycle assessment foreground system modeled are shown for the secondary and primary production routes in Supplementary Figures 4 and 5 respectively. We adapt gate-to-gate production inventories from ref.[3] (based on ref.[6]) and expand to cradle-to-gate boundaries for scrap supply from fossil infrastructure for the EAF production route. All flows outside of system boundaries are accounted for in the background systems. We assess three different types of steels occurring in fossil infrastrucuture according to ecoinvent: reinforcing steel (carbon steel, unalloyed steel), low-alloyed steel (<5% alloying elements), and chromium steel (stainless steel). The life cycle inventories are available in the data repository associated with this article.

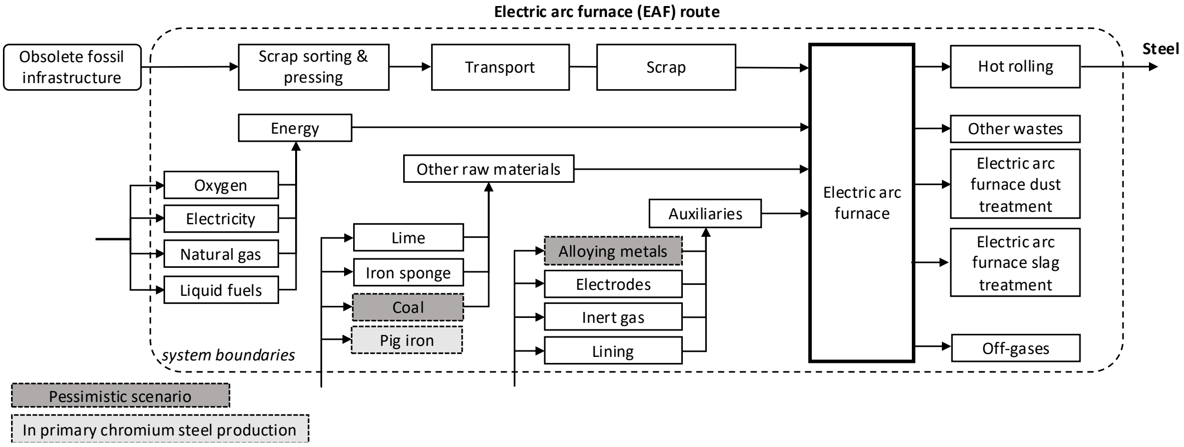

Supplementary Figure 4: **System boundaries for steel-making via electric arc furnaces.** The foreground system of secondary steel production includes energy, raw material, and auxiliary inputs as well as hot rolling, off-gases, and process waste treatments. Coal and alloying metal flows are set to zero in the optimistic scenario. Inventory adapted from ref.[3]

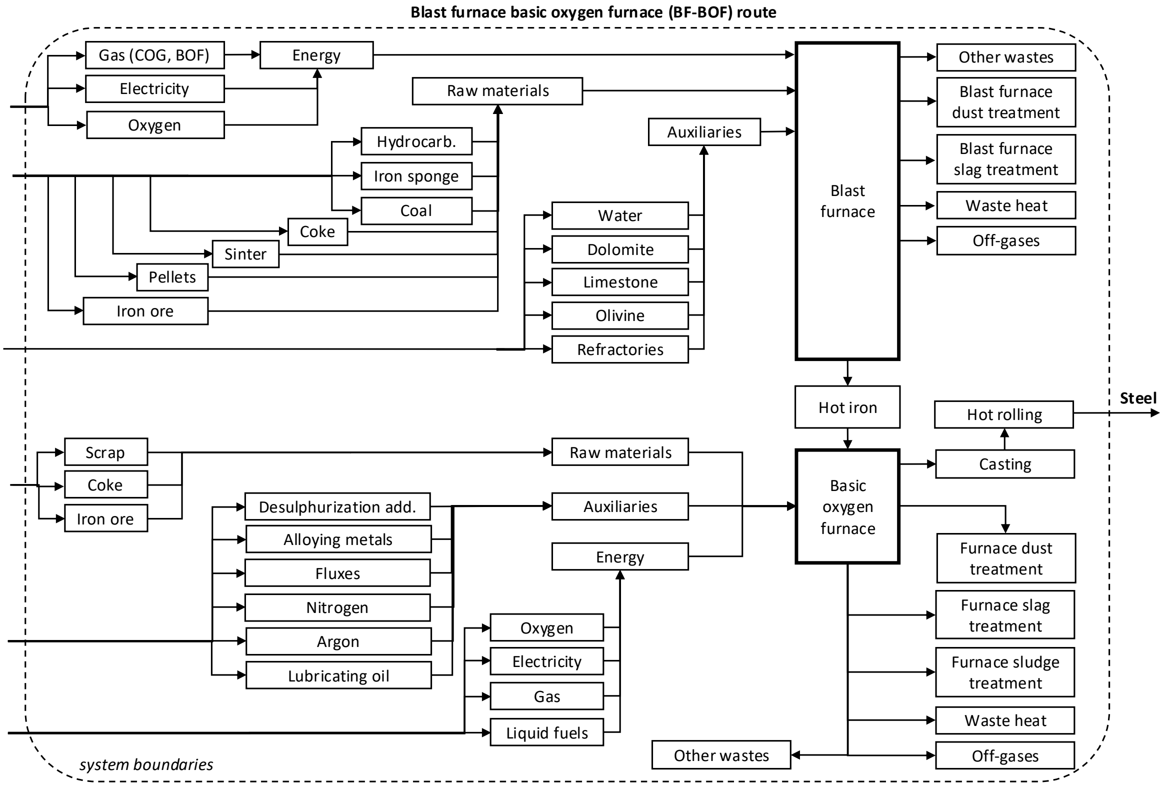

Supplementary Figure 5: **System boundaries for iron-making via blast furnace and steel-making via basic oxygen furnace.** The foreground system of primary steel production includes energy, raw material, and auxiliary inputs as well as casting, hot rolling, off-gas emissions, and process waste treatments. Inventory adapted from ref.[3] BF=blast furnace, COG=cogeneration, BOF=basic oxygen furnace.

Goal and scope for copper life cycle assessment

The functional unit is the amount of copper stored in the current fossil infrastructure produced. We compare the production of primary copper—following the hydrometallurgical and pyrometallurgical route—with the secondary production route using electrorefining. The pyrometallurgical production route is currently dominant (80% of primary production[2]), and will remain so when extrapolating fitted historical growth (78% in 2050, see Supplementary Fig. 6c).

The geographical scope is global and the temporal scope is the period 2025–2050. We model the globally weighted production average, for which we assume that the market shares of countries will remain constant (see Supplementary Fig. 6b). Moreover, we account for regional ore grade decline, taking values for the year 2010 documented in ref.[7] as the baseline and assume an annual ore grade decline of 0.0056 a^{-1} for Chile[8] and 0.0137 a^{-1} for all other countries[9] to project future ore grade (see Supplementary Fig. 6a).

For system boundaries of the copper production processes, see Supplementary Figures 7 and 8. The data sources and assumptions for the individual processes are as below.

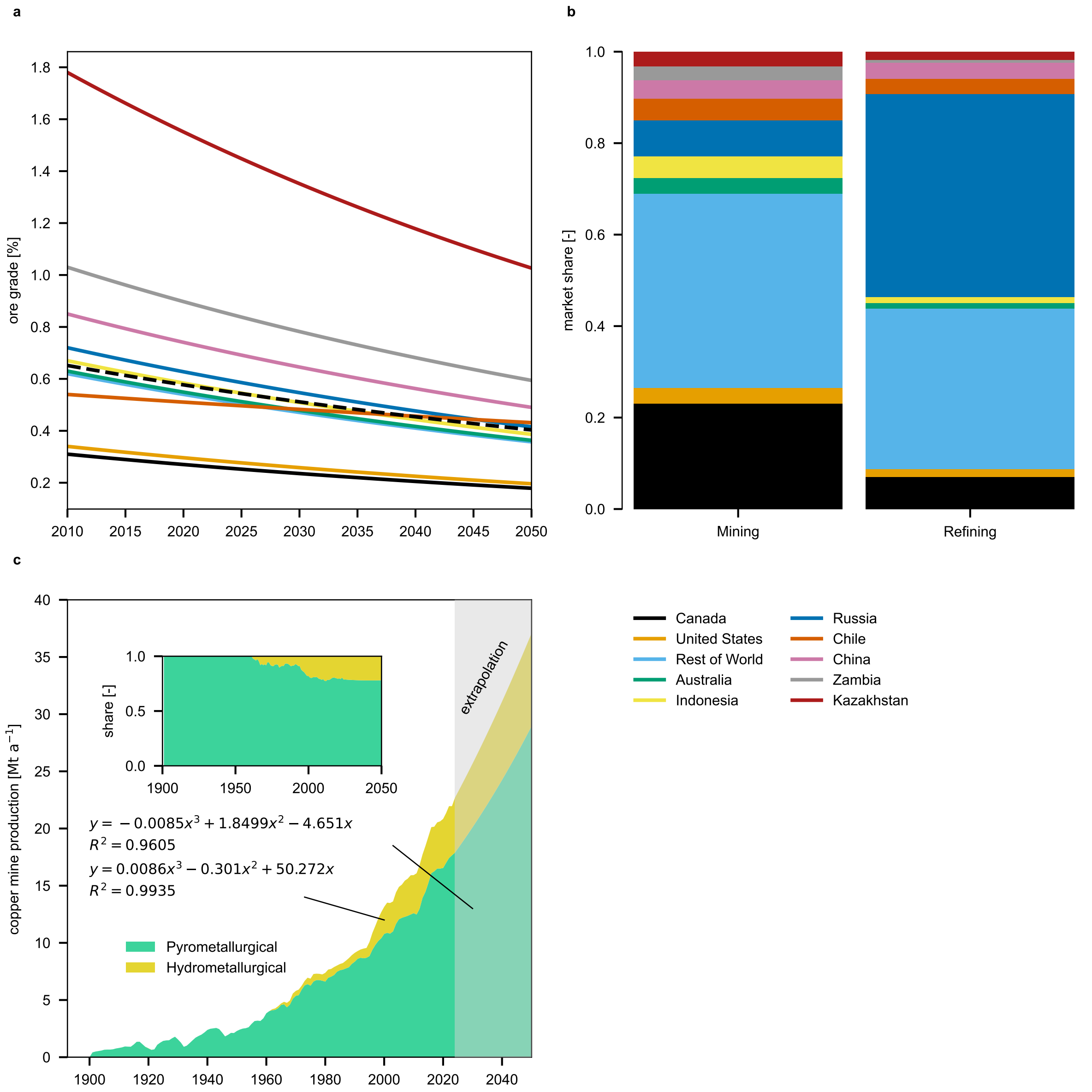

Supplementary Figure 6: **Ore grades and mining share by production type and country for copper.** **a**, Copper ore grade decline projections by country. The dotted black line indicates the globally weighted average. **b**, Copper mining and refining market shares by country (data source ref.[62]). **c**, Historical and extrapolated copper mine production by production route (historical data source ref.[2]). Mt=megatons, a=year.

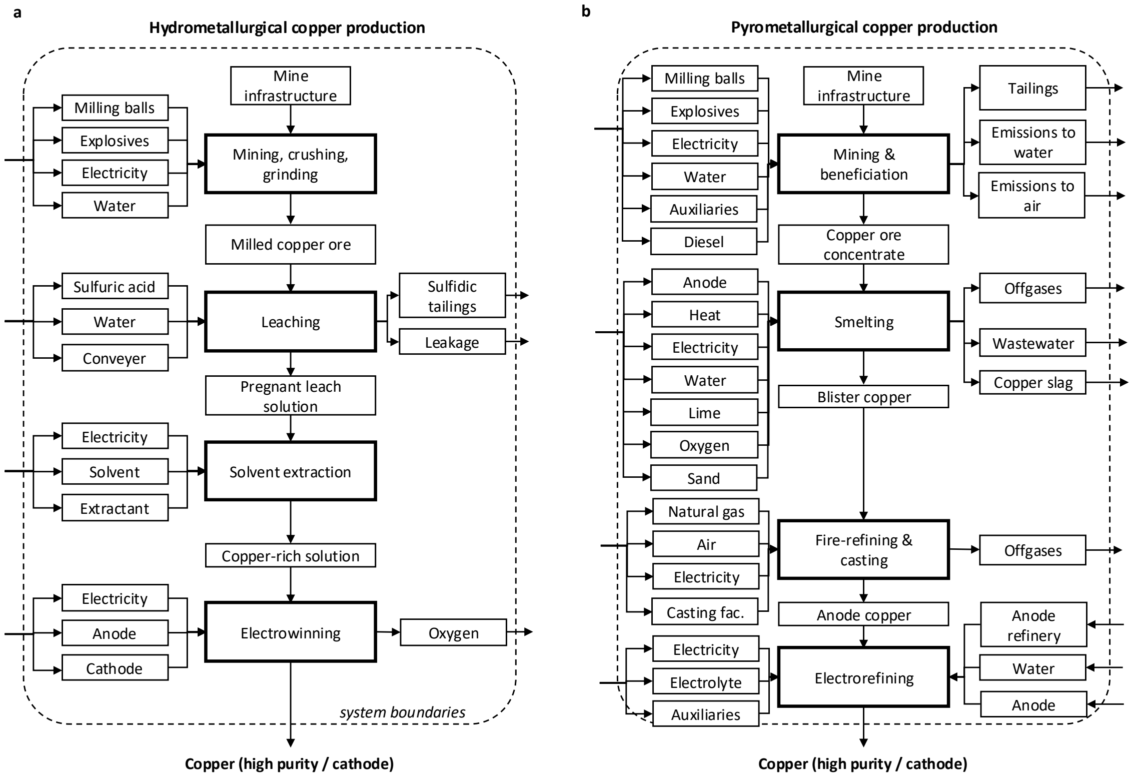

Supplementary Figure 7: **System boundaries for primary copper production.** The foreground system of copper production via (**a**) the hydrometallurgical and (**b**) pyrometallurgical route.

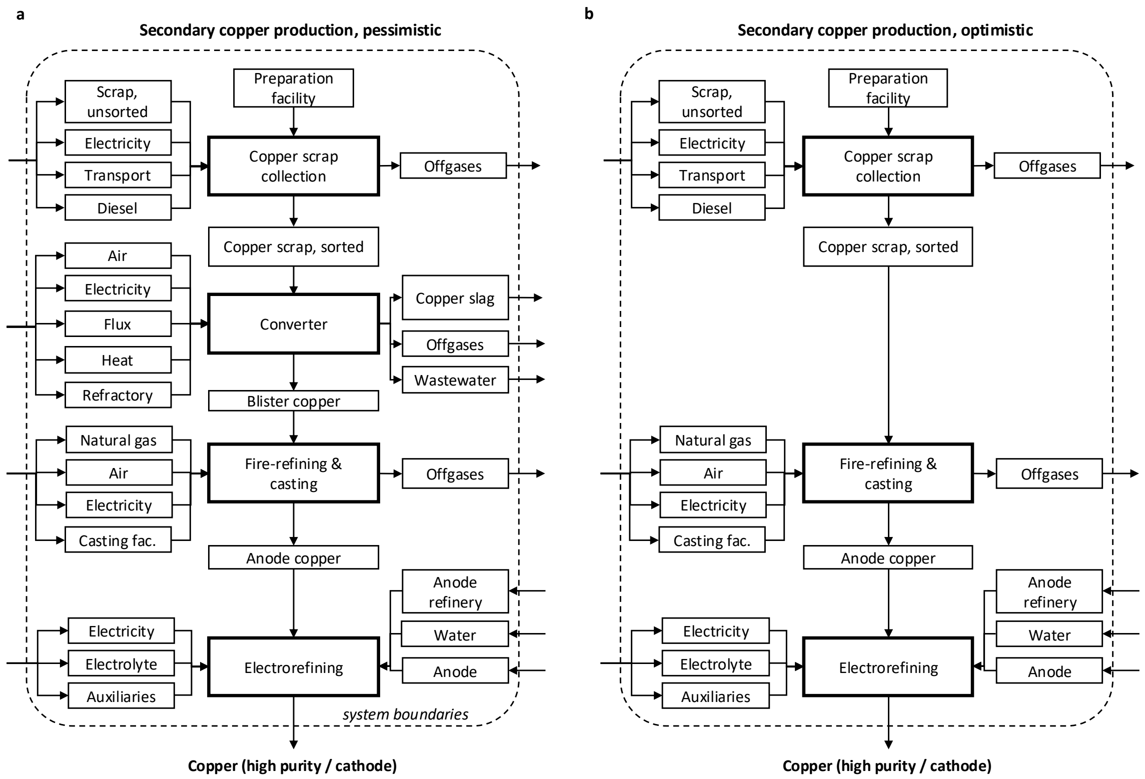

Supplementary Figure 8: **System boundaries for secondary copper production.** Flowcharts depict the foreground system of copper production via the secondary route. In the pessimistic case (**a**), low-grade copper scrap is treated in the converter to remove impurities. In the optimistic scenario (**b**), high-grade copper scrap is assumed to skip the converter step.

Hydrometallurgical route

Mining, crushing, and grinding. For mining copper ore, we assume the same input of explosive as in ecoinvent v3.11. The ore is then crushed and ground, requiring around 0.53 m^3 water per ton ore, 19.37 kWh electricity per ton ore, and 0.4 kg steel balls per ton ore (all means of mines in ref.[10]). The mine—85% open cast, 15% underground (estimate based on largest mines in ref.[2])—is assumed to produce 21 Mt a^{-1} of copper ore for 30 years. Ore composition is taken from ref.[11]

Leaching. The functional unit of leaching is 1 liter of pregnant leach solution (PLS). To obtain it, the ground ore is aggregated to heaps, sulfuric acid is added to leach copper from the ore, and the PLS is then collected with a liner system (ca. 3 g sulfuric acid per liter PLS). We account for the copper ore, the sulfuric acid solution, the conveyor infrastructure, as well as the waste tailings and emissions from leaked PLS. We further account for the area occupied under leach (mean of sites in ref.[10] : 4.9 $m^2 a\,ton^{-1}$ copper, translated with 0.0038 kg Cu per liter PLS). With 0.004 kg Cu recoverable per kg ore (mean of sites in ref.[10] and a Cu concentration of 0.0038 kg Cu per liter PLS (Supplementary Table 1), 1.22 kg ore is required per liter PLS). We assume that the same amount of 1.22 kg spent ore per liter PLS is managed as tailings after leaching. We further assume a leakage of 0.1% of the PLS to soil (for species in PLS, see Supplementary Table 2). For conveyor belts, we assume the same demand as in ecoinvent v3.11 (2.06×10^{-5} meter kg^{-1} Cu, translated with 0.0038 kg Cu per liter PLS). We adjust all inputs and outputs for decreased ore grades in the future according to Supplementary Equation (3) and (4) (see Supplementary Fig. 6a for assumed ore grades over time):

$$f_{c,j,cor} = \gamma_{c,j'} / \gamma_{c,j} \quad \forall c \in C, \forall j' = 2010, \forall j \in J \tag{3}$$

$$\mathrm{LCI}_{k,c,j,adjusted} = \mathrm{LCI}_{k,c,j} \cdot f_{c,j,cor} \quad \forall k \in K, \forall c \in C, \forall j \in J \tag{4}$$

with $\mathrm{LCI}_{k,c,j,adjusted}$ being the life cycle inventory for mining and benefication for all technosphere flows (K), countries (C) and years (J), $f_{c,j,cor}$ being the correction factor and $\gamma_{c,j}$ the country- and year-specific copper ore grade. The baseline year is 2010.

Supplementary Table 1: **Ranges of industrial copper leaching data (data source ref.[12]).**

Variable	Unit	Min	Max	Mean	Remark
Sulfuric acid addition	t H_2SO_4 per t Cu	1	3.5	2.56	[12]
Copper ore grade	%	0.26	1.16	0.66	[12]
Cathode copper capacity (C)	t a^{-1}	50000	366000	160833.3	[12]
Area under leach (A)	m^2	30000	1300000	736000	[12]
Occupation	m^{2}a t^{-1}	0.15	11.11	4.91	$= \frac{A}{C}$
Average leachable Cu (l)	%	0.23	0.88	0.53	[12]
Fraction recovered from PLS (f_{rec})	-	0.5	0.85	0.71	[12]
Recovered copper (R)	kg Cu (ion) per kg ore	1.22×10^{-3}	6.60×10^{-3}	3.98×10^{-3}	$= l \cdot f_{rec}$
PLS Cu concentration (c_{PLS})	kg Cu (ion) per liter PLS	2.50×10^{-3}	6.00×10^{-3}	3.75×10^{-3}	[12]
Ore demand	kg ore per liter PLS	0.65	2.46	1.22	$= \frac{R}{c_{PLS}}$

Supplementary Table 2: **Characterization of pregnant leach solution.**

Species	Range [g L^{-1}]	Mode [g L^{-1}]	Source
Cu^{2+}	2.5–6.0	3.8	[10]
H_2SO_4	6.0–10.0	8	[10]
Fe^{2+}	3.0–35.0	19	[10]
Al^{3+}	12.65–14.5	13	[50]
Mn^{2+}	1.47–1.68	1.5	[50]
Zn^{2+}	1.43–2.04	1.7	[51]
Mg^{2+}	12.98–13.25	13	[50]
SO_4^{2-}	95	95	[52]

Solvent extraction. The purpose of solvent extraction is to upgrade the PLS before electrowinning to yield a copper-rich electrolyte (from less than 15.5 g Cu per liter PLS to >45 g Cu per liter electrolyte[12]). For that purpose, copper ions are extracted from the PLS using an organic extractant (often kerosene), and the copper is then stripped from the organic phase.[12] The spent electrolyte (32–43 g Cu L^{-1}) is recycled and 2.2 liter PLS is required per liter electrolyte to replenish the copper content (mean of facilities in ref.,[12] see Supplementary Table 3). Furthermore, 0.08 kg kerosene and 0.04 kg extractant are needed per liter electrolyte when assuming a 1% loss and the electricity demand for mixing is 0.26 kWh per liter electrolyte (Supplementary Table 3).

Supplementary Table 3: **Ranges of industrial copper solvent extraction data (data source ref.[10]).**

Variable	Unit	Min	Max	Mean	Remark
PLS flowrate (F_{PLS})	m^3 h^{-1}	600	6041	1890	[10]
Spent electrolyte flowrate (F_{spent})	m^3 h^{-1}	426	1250	631	[10]
Organic phase flowrate ($F_{organic}$)	m^3 h^{-1}	600	2043	1238	[10]
Cu in PLS (c_{PLS})	g L^{-1}	2.5	15.5	6.7	[10]
Cu in electrolyte (c_{elec})	g L^{-1}	45	54	50	[10]
Cu in spent electrolyte (c_{spent})	g L^{-1}	32	43	37	[10]
Cu in feed (c_F)	g L^{-1}				$= \frac{c_{PLS}(F_{PLS}-F_{spent})+c_{elec}F_{spent}}{F_{PLS}}$
Fresh PLS per electrolyte (f_{fPLS})	L L^{-1}	1.0	3.9	2.2	$= \frac{c_{ele}}{c_F}$
Organic phase per fresh PLS (f_{org})	L L^{-1}	0.4	35.0	6.3	$= \frac{F_{organic}}{F_{PLS}-F_{spent}}$
Organic phase loss per fresh PLS ($f_{org,loss}$)	L L^{-1}	0.004	0.350	0.063	$= 0.01 \cdot f_{org}$
Extractant volume fraction ($f_{vol,extr}$)	-	0.16	0.34	0.23	[10]
Loss extractant per fresh PLS (l_{extr})	L L^{-1}	0.001	0.119	0.020	$= f_{vol,extr} \cdot f_{org,loss}$
Loss solvent per fresh PLS (l_{solv})	L L^{-1}	0.003	0.231	0.043	$= f_{org,loss} \cdot f_{vol,extr}$
Power demand (P)	kW	21	69	50	[10]
Electricity demand, aquatic and organic flow (P_{org})	kW m^{-3}	0.003	0.249	0.109	$= \frac{P}{(F_{organic}-F_{spent})f_{org}}$
Electricity demand, electrolyte (P_{ele})	kW m^{-3}	0.003	0.681	0.264	$= P_{org} \cdot f_{fPLS}$

Electrowinning. Pure copper is won at the cathodes by reducing the Cu^{2+} ions in the electrolyte by applying a voltage between a lead anode and a stainless steel cathode.[13]

Pyrometallurgical route

Mining and beneficiation. Inputs and outputs for the mining and beneficiation process of copper ore concentrate are adjusted values from the ecoinvent v3.9.1 process.[7] The electricity input is relinked to the respective mix in each country for the respective year, and all technosphere inputs are adjusted for declining ore grade using the correction factor as in Supplementary Equations 3 and 4.

Smelting. Inputs and outputs for copper concentrate smelting are based on the *smelting of copper concentrate, sulfide ore* activity in ecoinvent v3.11 for the respective countries. For copper mining countries that are not represented in ecoinvent, we adjust the Rest of World dataset for the local electricity mix.

Fire-refining and casting. Fire-refining utilizes a rotary anode furnace to remove contaminants from the copper.[14] We assume an air input of $9\times10^{-5}\,m^3$ per kg anode copper, based on 0.5 h oxidation per cycle at $50\,m^3\,h^{-1}$ and for 270 ton copper anode production per cycle. The furnace is fired with natural gas of a flow rate of $20\,m^3\,min^{-1}$ for 3 h per cycle.[14] The furnace is assumed to have a lifetime of 50 years at 130,000 tons annual production. We calculate the carbon dioxide, methane, and dinitrogen monoxide emissions from natural gas emission factors and base the sulfur dioxide emission (0.0009 kg $SO_2\,kg^{-1}$ anode copper) on the assumption that the sulfur in off-gases is equal to the difference of sulfur in copper before and after fire-refining (0.05 m% before and 0.005 m% after[14]). The electricity demand for casting is assumed to be the same as in brass casting activity in ecoinvent v3.11 (0.0197 $kWh\,kg^{-1}$).

Electrorefining. Electrorefining is an electrochemical process that forces copper at the anode into solution by applying a voltage. Pure copper aggregates at the cathode, usually a steel plate. The inputs needed for electrorefining are copper at the anode, an electrolyte, additives (proteins), grain-refining agents, the cathode, electricity, and the anode copper refinery. The anodic copper demand per kg cathodic copper is 1.005 kg (assuming a 0.5% loss). The copper-rich electrolyte (density 1.25 $kg\,m^{-3}$[15]) has an estimated bleed flow of $0.15\,m^3$ per ton cathodic copper, thus 0.0001875 kg electrolyte per kg cathodic copper is required for replacement. Further, glue (proteins) is required as a leveling agent at a rate of 0.12 kg per kg cathodic copper.[16] Grain-refining agents are added, more precisely around 150 mg of thiourea and 7.5 mg of chloride per kg cathodic copper.[16] Cathodes are usually $1\,m^2$ stainless steel plates of 1 mm thickness. For the assumed factory size, usually around 50,000 cathodes are used[16] with a lifetime of 10 years.[17] The electricity demand for voltage formation is 0.291 kWh per kg cathodic copper.[16] Regarding the anodic copper refinery, we assume an annual production of 270,000 tons and a lifetime of 30 years. Anode slime is usually further treated in other metal extraction processes, which is why we treat anode slime treatment as burden-free here.

Secondary production

Copper scrap collection and sorting. We assume that copper scrap is burden-free when collected from fossil infrastructure and requires 382.5 kilometers of transport to the scrap preparation facility (same as in ecoinvent v3.11, transport mode split as in *market for copper scrap, sorted, pressed*). We do not perform an estimation of copper scrap distances to regional copper recycling plants as we do for steel since a comprehensive dataset on the location and capacity of local copper recycling plants is missing. We do, however, perform a sensitivity analysis on the copper scrap transport distance (see Supplementary Fig. 9) and find that the climate impact and externality cost increase by less than 7% and less than 9% respectively when increasing the transport distance by 50%. For sorting, we assume the same electricity, diesel, and facility demand per kg copper as for iron scrap sorting in ecoinvent v3.11.

Converter. In the case of high-grade scrap, the converter step can be skipped and the scrap can be directly fire-refined and cast (optimistic scenario). We test a pessimistic scenario, in which low-grade copper scrap is treated in a converter to remove impurities. We assume that 1.2 kg scrap per kg copper is required (range in ref.[18]). The energy demand is 9.1 MJ heat per kg copper—which we assume is supplied with natural gas—and 1.1 kWh per kg copper of electricity.[19] Furthermore, oxygen is required for combustion at a rate of $30{,}000\,m^3\,h^{-1}$ at an assumed capacity of $330\,ton\,d^{-1}$, yielding $2.18\,m^3$ per kg copper. We also account for refractory demand (magnesium oxide, 4.7 g per kg copper; conservative estimate from ref.[18]) and additional fluxes of 74 g lime per kg copper and 63 g silica per kg copper.[19] Offgas emissions by the converter are taken from ref.[19] and carbon dioxide emissions from natural gas combustion are calculated with an emission factor of 1.98 $kg\,CO_2\,m^{-3}$ (heating value 43 MJ per m^3 natural

gas).

Fire-refining and casting. The inputs and outputs are the same as in the fire-refining and casting process of the pyrometallurgical route, except that the copper input is copper scrap in the optimistic scenario and anodic copper from the converter in the pessimistic scenario.

Electrorefining. All inputs and outputs are the same as in the electrorefining process of the pyrometallurgical route.

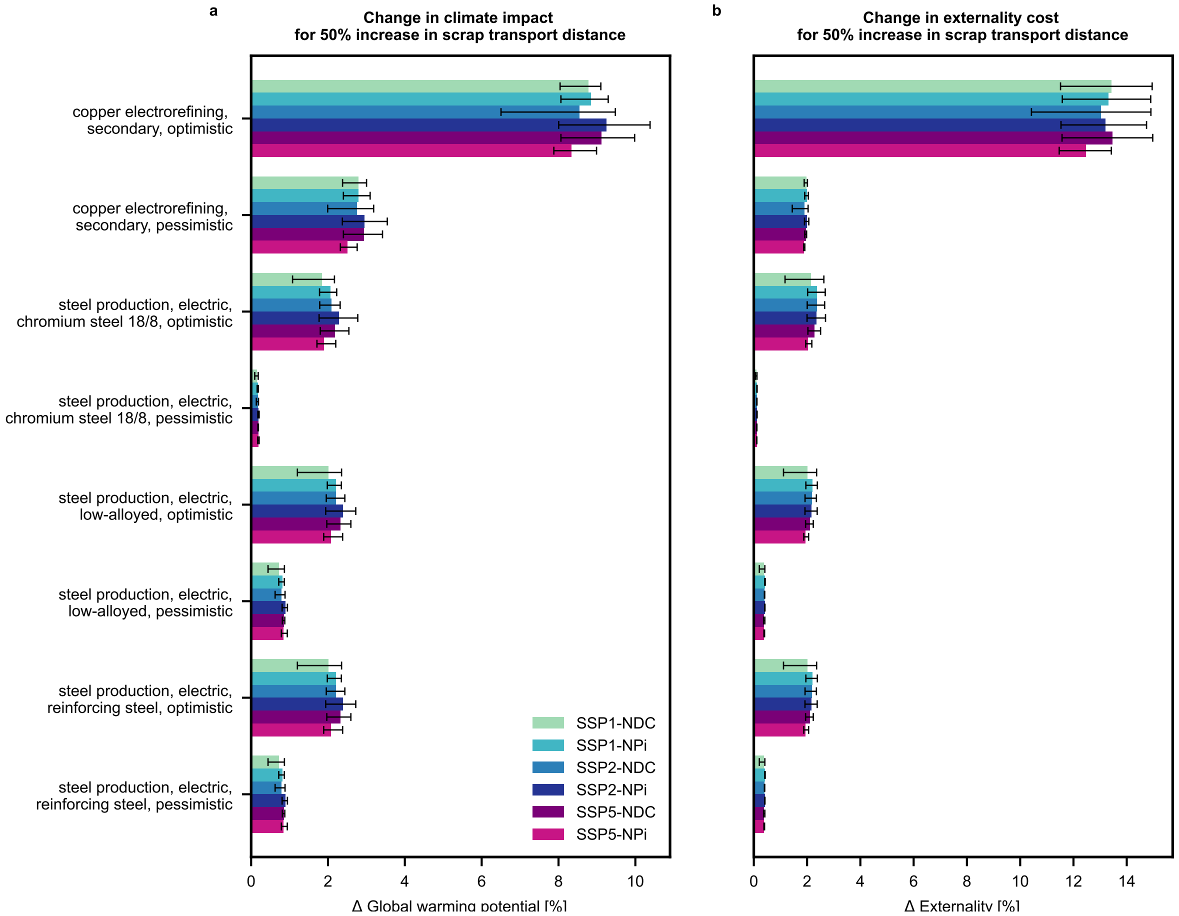

Supplementary Figure 9: **Life cycle impact sensitivity to scrap transport**. Sensitivity of (**a**) climate impact and (**b**) externality cost of secondary steel and copper production to scrap transport distance. Error bars show minimum and maximum sensitivity for possible years of recycling (2025 to 2050). SSP=Shared socio-economic pathway, NPi= National policies implemented, NDC=National determined contributions.

Scrap transport estimation

We estimate a globally representative steel scrap transport distance from fossil infrastructure to recycling plants by weighing distances within countries and between countries. To yield a representative transport distance within a country, the distance of each fossil infrastructure to the closest recycling plant (assuming it to be the most economical option) is calculated and weighed by the mass share of the total country steel waste stock in fossil infrastructure (see Supplementary Eq. 5). The representative country distances are then weighted by the share of country stocks of scrap according to supplementary equation (5) and (6).

$$D_c = f_r \frac{1}{s_{c,total}} \sum_{n \in N_c} d_n \cdot s_n \quad \forall c \in C \tag{5}$$

$$D_{GLO} = \frac{1}{s_{total}} \sum_{c \in C} D_c \cdot s_{c,total} \tag{6}$$

D_c is the representative scrap transport distance in country c (an element of all assessed countries C), n is a fossil infrastructure in the set of all the country's fossil infrastructure N_c, d_n is the distance of n to the closest recycling plant (the straight line between two points on a globe), and s_n is the steel scrap stock in n. The route factor f_r corrects for the fact that real transport distances are longer than the ideal distances (straight line) and is assumed to be 1.5. The total scrap stock in country c is $s_{c,total}$, and s_{total} is the global total scrap stock in fossil infrastructure. D_{GLO} is the global representative scrap transport distance, which is 481 km if the scrap is transported to the closest recycling plant. This is in good agreement with the 537 km that ecoinvent v3.9.1 assumes for sorted and pressed iron scrap in Rest of World. The transport mode split is the scrap stock weighted average of countries (assuming global mode split, in those instances where country-level data is missing). The life cycle impacts of steel are not sensitive to the scrap transport distance, e.g. climate impact and externality costs increase by less than 3% across scenarios, steel types, and years when scrap transport distance is increased by 50% (see Supplementary Fig. 9).

For copper, scrap transport distances to recycling plants are not estimated because no comprehensive dataset is publicly available for copper recycling plants and their location. We assume the copper scrap distances to be the same as in ecoinvent v3.11 (536.6 km) and perform a sensitivity analysis (see Supplementary Fig. 9). The sensitivity of the life cycle impacts to the scrap transport is higher than for steel (in the optimistic scenario), but overall low: Increasing the transport distances by 50% increases climate impact and externality costs by less than 7% and less than 9% respectively.

Steel production efficiencies

Different steel production efficiencies are assumed for investigated scenarios. Supplementary Figure 10 shows process inputs (e.g. coal, natural gas, electricity, ferromanganese), carbon emissions, and carbon capture relative to process flows in SSP1-NDC. Efficiency gains target the blast furnace, basic oxygen furnace (primary) route, and the electric arc furnace (secondary) route. Values are taken from Premise.[3]

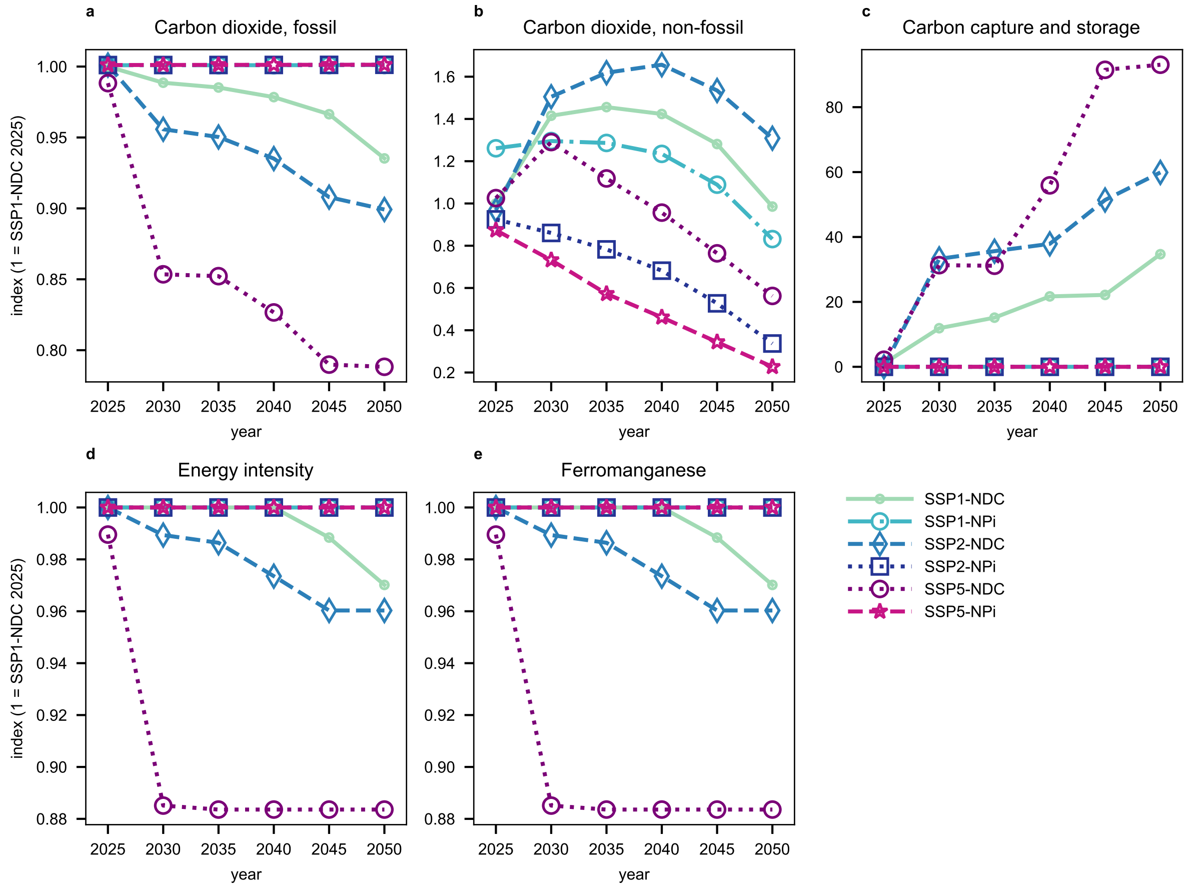

Supplementary Figure 10: **Process efficiencies**. Efficiencies of primary and secondary steel-making regarding (**a**) fossil carbon dioxide, (**b**) non-fossil carbon dioxide, (**c**) carbon capture and storage, (**d**) the energy intensity of steel production, and (**e**) ferromanganese input in depending on the scenario. All input amounts are normalized to SSP1-NDC 2025. Efficiencies are taken from Premise.[3] SSP=Shared socio-economic pathway, NPi= National policies implemented, NDC=National determined contributions.

Inventory adjustments for electric arc furnace hexavalent chromium content in slag and dust

Hexavalent chromium (Cr^{6+}) creates adverse health effects in humans and other organisms[20,21] and can leak from electric arc furnace slag and dust into the environment. The content of Cr^{6+} in slag and dust can vary significantly, which is why we modify the standard ecoinvent inventory to capture the range of potentially leaking Cr^{6+} by selecting low and high values found in literature for the optimistic and pessimistic scenarios respectively.

We also adjust Cr^{6+} release in electric arc furnace slag treatment in ecoinvent, as ecoinvent likely overestimates Cr^{6+} leaching. Measurements of Cr^{6+} content in electric arc furnace slag from 58 steel mills[22] yield a mean of 1.2 mg Cr^{6+} per kg slag. We take the 2.5th percentile assuming a log-normal distribution as the optimistic value (0.14 mg Cr^{6+} per kg slag) and the 97.5th percentile as the pessimistic value (67.17 mg Cr^{6+} per kg slag)—which is significantly lower than 7.05×10^3 mg Cr^{6+} kg^{-1}, assumed in ecoinvent. We assume that half the Cr^{6+} is emitted to groundwater and half to surface water.

The chemical composition of electric arc furnace dust can vary, depending on the chemical composition of steel scrap and operating conditions.[23] Due to missing representative literature data on Cr^{6+} release from dust, we adopt the uncertainty range indicated in *treatment of electric arc furnace dust, residual material landfill* (RoW) taken from ecoinvent v3.9.1. As an optimistic scenario, we assume the 2.5th

percentile of Cr^{6+} release, yielding 2.07×10^{-4} kg and 5.03×10^{-5} kg per kg dust to groundwater and surface water respectively. As a pessimistic scenario, we take the 97.5th percentile of the emission uncertainty, yielding 3.94×10^{-3} kg and 1.46×10^{-3} Cr^{6+} kg per kg dust to groundwater and surface water respectively.

Renewable energy system inventories

Inventories for solar photovoltaic energy systems are taken from ecoinvent v3.9.1, more precisely *photovoltaic plant construction, 570kWp, multi-Si, on open ground (GLO)* and *photovoltaic slanted-roof installation, 3kWp, single-Si, panel, mounted, on roof (RoW)* (including electrical installation, maintenance, mounting system, and inverter[7]). For the module, we use updated single-Si PV module datasets from ref.[24] For wind, we also use inventories from ecoinvent v3.9.1, namely *wind turbine construction, 4.5MW, onshore (GLO)* for onshore wind, and *wind power plant construction, 2MW, offshore, moving parts (GLO)* and *wind power plant construction, 2MW, offshore, fixed parts (GLO)* for offshore wind. We further assess impact and externality cost reductions for three types of electrolyzers and power transmission infrastructure. The inventories for proton exchange membrane (PEM), alkaline electrolysis cells (AEC), and solid oxide electrolyzer cells (SOEC) electrolyzers are taken from Premise.[3] The power transmission infrastructure dataset is taken from ecoinvent v3.9.1 (*transmission network construction, electricity, medium voltage (RoW)*). All inventories are available in the data repository.

Supplementary Methods 5: Steel and copper production cost

Secondary steel production is competitive with primary steel production (see Supplementary Fig. 11) and can even be slightly cheaper (–0.3%, –5.1%, and –6.3% less production cost for reinforcing, low-alloyed, and chromium steel respectively on plant average).[25]

Secondary copper production is substantially cheaper than primary copper production, although costs vary with scrap quality and production route respectively (Supplementary Table 4). Producing virgin copper costs between 830 and 5,600 USD via the hydrometallurgical route depending on the country and between 1,460 and 4,500 USD via the pyrometallurgical route (years 2010, 2011). The globally weighted production cost in 2008 to 2010 was around 2,600 USD.[26] Recycling costs for copper from e-waste are between 5,500 and 11,700 USD, between 4,200 and 25,900 USD from scraped car recycling, and between 1,000 and 2,300 USD from copper cable recycling. As copper in fossil infrastructure is mostly present in cables, we assume a secondary copper production cost of 2,300 USD.

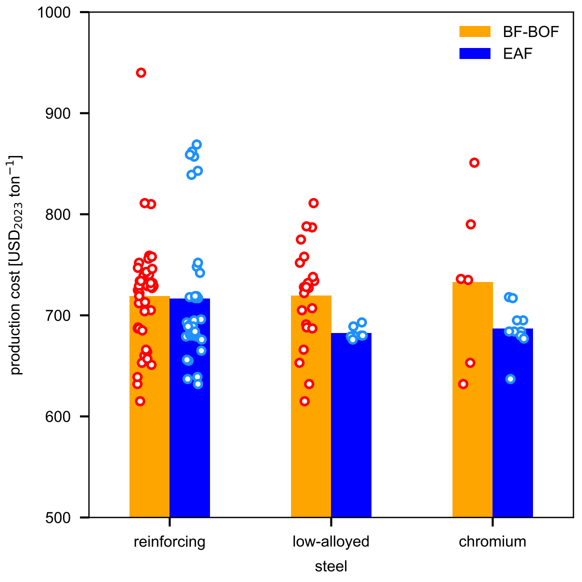

Supplementary Figure 11: **Steel production cost for blast furnace basic oxygen furnace route (BF-BOF) and electric arc furnace route (EAF) in the year 2021**. The production cost of 125 individual steel plants in 63 countries (red and blue dots). Bar shows the plant average production cost. Data taken from ref.[25] USD_{2023}=US Dollars in 2023 equivalent.

Supplementary Table 4: **Costs for different copper production processes and routes.**

Activity	USD ton^{-1} min	max	mode	Remark
Virgin mining hydrometallurgical	830	5600	3215	[53]
Virgin mining pyrometallurgical	1462	4500	3346	[53] mode,[26] adjusted more inflation 2014–2023
Urban mining e-waste	330	1572	1572	[53]
Urban mining cables	500	2250	2250	[53]
Urban mining EoL vehicles	9	463	463	[53]
Producing Cu anode from raw material	11	457	354	[54] including credits, adjusted for inflation 2016 to 2023
Producing Cu cathode from anode	22	270	57	[54] including credits, adjusted for inflation 2016 to 2023
Total hydrometallurgical	863	6327	3626	mining + Cu anode + Cu cathode
Total pyrometallurgical	1495	5227	7030	mining + Cu anode + Cu cathode
**Total primary production average**	**1369**	**5447**	**6349**	80% pyrometallurgical, 20% hydrometallurgical
**Total cable recycling**	**533**	**2977**	**2661**	urban cable mining + Cu anode + Cu cathode

Supplementary Methods 6: Steel use in photovoltaic systems

Aluminum (Al) is used in PV mounting systems to connect the module to a surface (e.g. roof, ground). We test the environmental effect of using steel instead of Al for mounting the module. To estimate the steel needed for this purpose we assume two L-shaped steel profiles with 0.03 m height and width, a thickness of 0.003 m, and a length of 1.08 m to mount a module with the dimensions of 1 m$\times$1.85 m. With a steel density of 7,860 kg m^{-3}, this yields 1.53 kg steel per m^2 or 0.54 kg steel per substituted kg Al (Al intensity taken from ecoinvent v3.9.1 for slanted-roof PV; 2.84 kg Al per m^2). For open-ground PV systems, we substitute Al with the same factor of 0.54 kg steel per kg Al.

Could the use of steel in mounting systems increase PV installation cost? We estimate the hardware cost and installation cost changes for utility-scale PV resulting from switching from Al mounting systems to steel mounting (see Supplementary Fig. 12). We test substitution of 3.98 kg Al m^{-2} (at 200 Wp m^{-2}) at a price of 3.0 USD per kg Al (standard deviation 20%, normal distributed) with 2.15 kg m^{-2} steel at a price of 0.7 USD kg^{-1} (standard deviation 20%, normal distributed). We then run a Monte Carlo simulation (1,000 runs) for the hardware cost changes resulting from switching materials (see costs in Supplementary Fig. 12c and d), resulting in reduced mounting system costs of 50 USD (kWp)$^{-1}$ (21–84 USD (kWp)$^{-1}$, 95% confidence interval) or 3.0 % (1.2–5.0%) of the total installation cost (1,685 USD (kWp)$^{-1}$, average of 33 countries[27]).

Due to missing data on labor costs for steel mounting system installation, we assume that mounting accounts for 40% (10% standard deviation) of the total mechanical installation costs (55 USD (kWp)$^{-1}$, standard deviation 17 USD (kWp)$^{-1}$ within 33 countries;[27] Supplementary Fig 12b). We further make a conservative assumption that installation costs for steel mounting systems are double the costs of Al systems, which would increase costs by 21 USD (kWp)$^{-1}$ (8–47 USD (kWp)$^{-1}$) (Supplementary Fig. 12b).

The net cost benefits of PV using steel mounting systems might thus be 28 USD (kWp)$^{-1}$ (–9–66 USD (kWp)$^{-1}$) or 1.7% (–0.5–3.2%) of the total installation cost (see Supplementary Fig. 12a and e). This estimated cost benefit should be viewed with caution in light of the lack of data collected from practitioners. Overall, PV installation costs are likely not sensitive to the type of mounting system used.

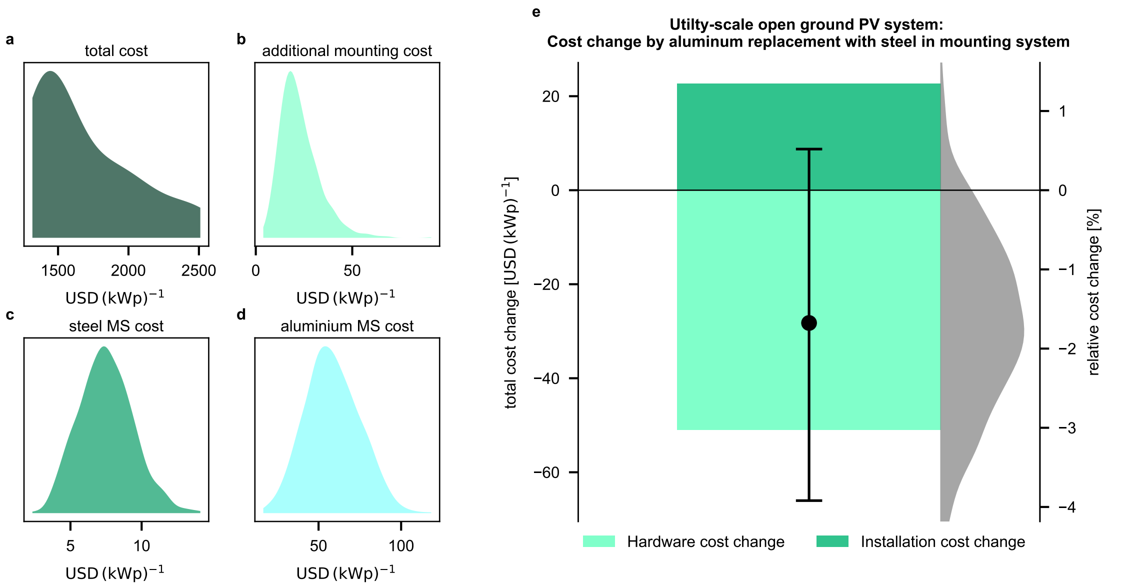

Supplementary Figure 12: **Photovoltaic installation cost changes through steel mounting systems utilization.** We show assumed cost distributions for key cost parameters: **a**, total cost distribution of 33 countries,[27] (**b**), estimated additional cost for mounting, (**c**) estimated cost for steel mounting system (MS) and (**d**) aluminum MS. **e**, Estimated total and relative change in hardware and installation costs of utility-scale open ground PV, including probability density in gray. Error bar shows 95% confidence interval of net cost change measured with Monte Carlo simulation of n=1000 runs for possible hardware and installation costs (**b–d**). Labor costs for mounting are assumed to be doubled. USD_{2023}=US Dollars in 2023 equivalent, kWp=kilowatt peak capacity, PV=photovoltaic.

Supplementary Methods 7: Material intensities of fossil infrastructure and their uncertainty

Material intensities

Supplementary Figure 13 shows material intensities for fossil infrastructure assessed based on ecoinvent v3.9.1.[7] Detailed uncertainty values can be found in the data repository of this study.

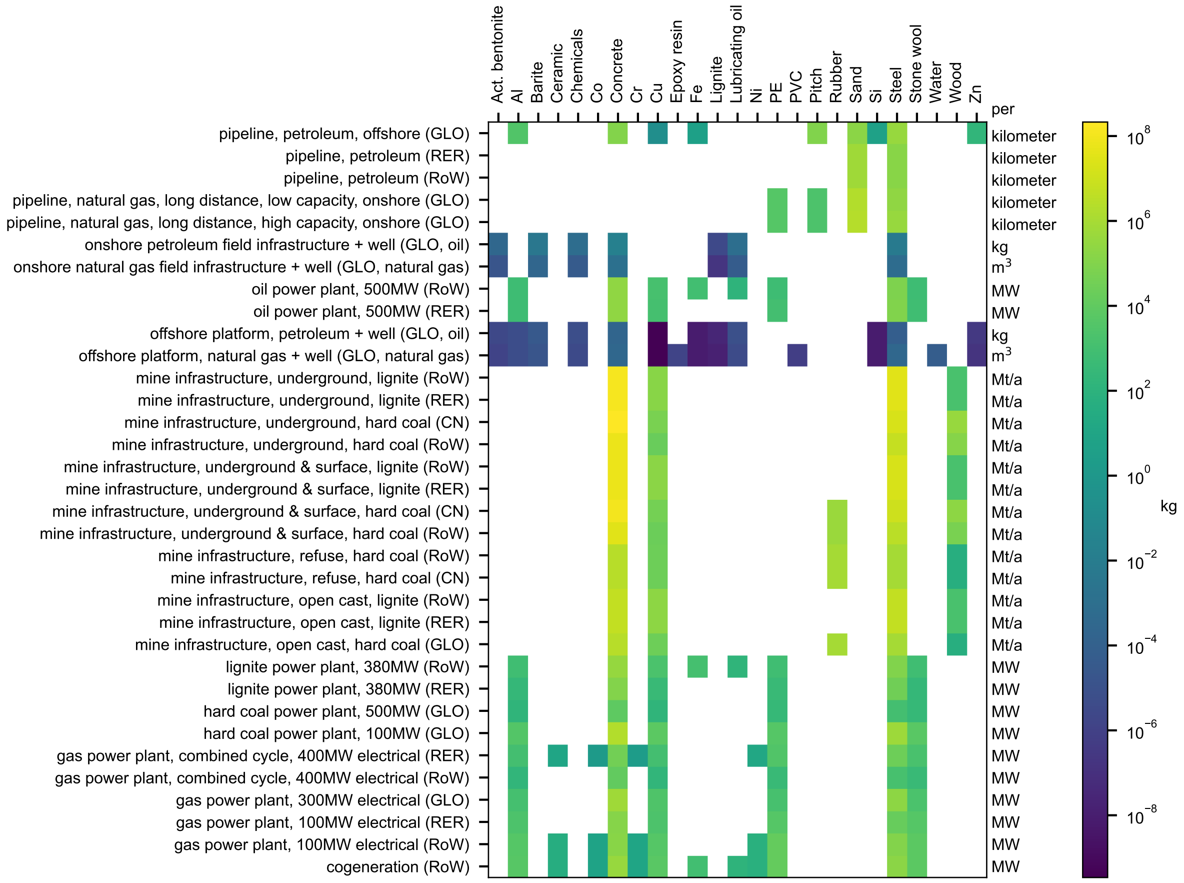

Supplementary Figure 13: **Normalized material intensities of fossil infrastructure.** Oil and gas rig material intensities are shown normalized to kg and cubic meters of oil and gas extracted respectively, coal mine intensities are normalized to Mt a^{-1} coal extraction capacity, power plant material intensities are normalized to power capacity, and pipelines material intensities per kilometer length. Brackets indicate geographical region (GLO=global, RER=Europe, RoW=Rest of World, CN=China) and fuel type in case of oil and gas rigs. Abbreviations of materials are Cr for Chromium, PE for polyethylene, Ni for Nickel, Co for Cobalt, PVC for polyvinyl chloride, Si for Silicon, Zn for Zinc, Cu for Copper, Fe for Iron, and Al for Aluminum. MW=megawatt, Mt=megatons, a=year.

Uncertainties

Multiple sources of uncertainties exist regarding material stocks in fossil infrastructure. 1) Uncertainties arise from the geographical and temporal variability of infrastructure, which is modeled with log-normal distributions for each material intensity in ecoinvent. The standard deviation for each material intensity can be accessed in the data repository. 2) Some material intensities, such as wood and concrete, are reported as volume and require translation to mass using densities. The density uncertainties for these

materials are shown in Supplementary Table 5. 3) Fossil infrastructure is classified differently in ecoinvent and in Global Energy Monitor (GEM) databases. This generates linking uncertainty in selecting the representative material intensity for some infrastructure types. We model these uncertainties by a uniform distribution covering the range between minimum and maximum possible material intensities arising from linking choices (see data repository for values). For example, a power plant with 350 MW capacity can either be assigned material intensities of a 100 MW or 500 MW power plant. In the case of this linking choice, we adopt a threshold (e.g. 300 MW) to assign the infrastructure to the more representative infrastructure class. Threshold values are shown in Supplementary Table 6.

Supplementary Table 5: **Volumetric density parameters and their uncertainty for selected materials.**

**Material**	**Uncertainty type**	**Min [kg m^{-3}]**	**Mode [kg m^{-3}]**	**Max [kg m^{-3}]**	**Sigma**	**Remark**
plywood	uniform	4.00×10^2	5.50×10^2	7.00×10^2		assumed
sawnwood, hardwood, raw, dried (u=20%)	uniform	3.50×10^2	5.60×10^2	7.70×10^2		assumed
concrete, normal strength	normal		2.35×10^3		6.96×10^1	ecoinvent v3.9.1
unreinforced concrete, 15MPa	normal		2.35×10^3		6.96×10^1	ecoinvent v3.9.1
Wood*	uniform	3.50×10^2	5.60×10^2	7.70×10^2		assumed
Concrete*	normal		2.35×10^3		6.96×10^1	ecoinvent v3.9.1

*aggregated material category

Supplementary Table 6: **Threshold values for linking infrastructure classes.**

**Infrastructure**	**Threshold**	**Interval**	**Unit**	**Variable**
hard coal power plant	3.00×10^2	$[1.00, 5.00]\times10^2$	MW	capacity
gas power plant	2.00×10^2	$[1.00, 3.00]\times10^2$	MW	capacity
pipeline, natural gas, long distance, onshore	0.95×10^6	$[0.8, 1.1]\times10^6$	norm m^3 h^{-1}	throughput
pipeline, natural gas, long distance, onshore	1.085*	[0.950, 1.219]	m	diameter

*if throughput data missing

Furthermore, some infrastructure classes in Global Energy Monitor (GEM) databases[28] are not present in ecoinvent, such as combined underground and surface coal mines (open pit) as well as refuse coal mines (surface coal wastes at coal mines). We model the material intensities of combined underground and surface coal mine as the mean of an underground and surface coal mine, and the refuse coal mine as an open pit mine.

The material intensity of oil and gas extraction infrastructure is sensitive to borehole length. As the GEM database does not indicate the borehole length of individual wells, we take 3,000 m being the mean of 17,540 oil wells in the Bakken Formation[29] and account for possible other borehole lengths in the linking uncertainty (assuming a minimum of 1,000 m and a maximum of 5,000 m borehole length, being the range indicated in ref.[29]).

We propagate all uncertainty by running a Monte Carlo simulation (n=500), generating a distribution of possible material stocks for each infrastructure.

Supplementary Methods 8: Life cycle impact indicators

We translate midpoint indicators—usually emissions—into damage to human health and ecosystems as well as pressures on natural resources (endpoint indicators) according to the ReCiPe Framework 2016 v1.1[30] (see Supplementary Fig. 14, 15, and 16). Human health-related environmental impacts are converted to human health impacts in units of Disability Adjusted Life Years (DALYs), ecosystem-related midpoint indicators are converted into ecosystem damage in units of species years lost, and fossil and mineral extraction into surplus cost potential in units of US dollars.

All life cycle impact assessment methods used to calculate midpoint indicators and their respective characterization factors are available in the data repository.

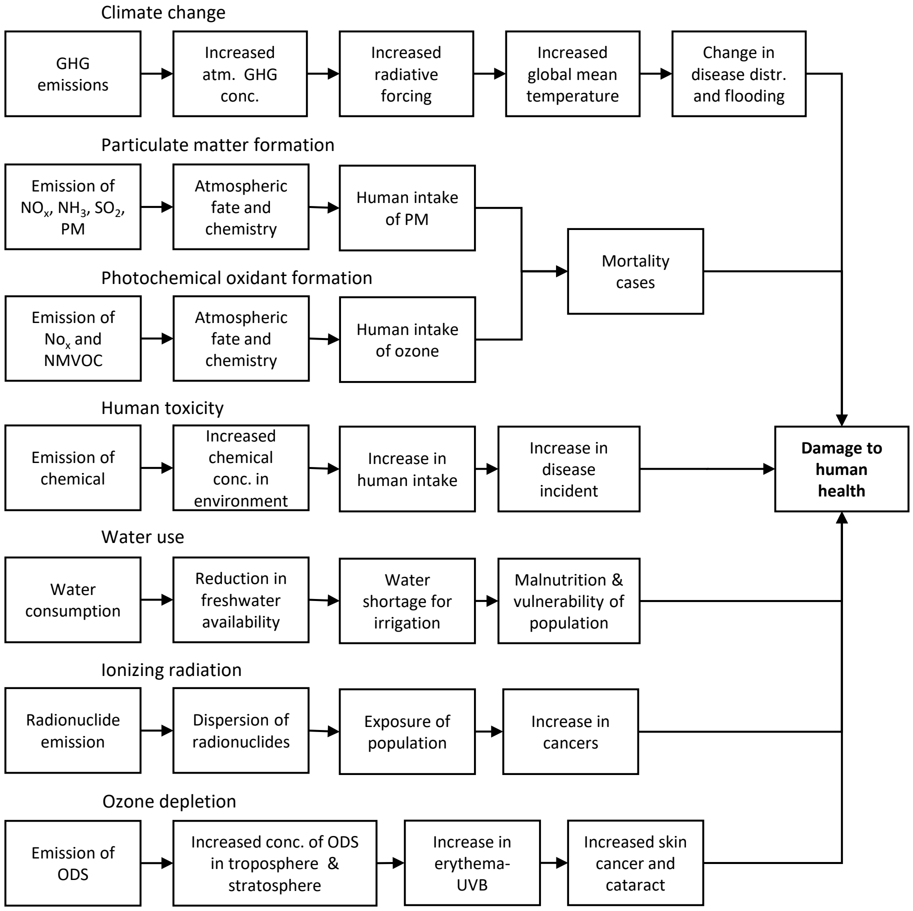

Supplementary Figure 14: **Cause-and-effect chain of midpoint indicators to human health impact.** Figure based on ref.[74] GHG=Greenhouse gas emissions, PM=Particular matter, NMVOC=Non-methane volatile organic compounds, ODS=Ozone depleting substances, UVB=Ultraviolet B radiation, NH_3=ammonia, SO_2=sulfur dioxide, NO_x=nitrogen oxides.

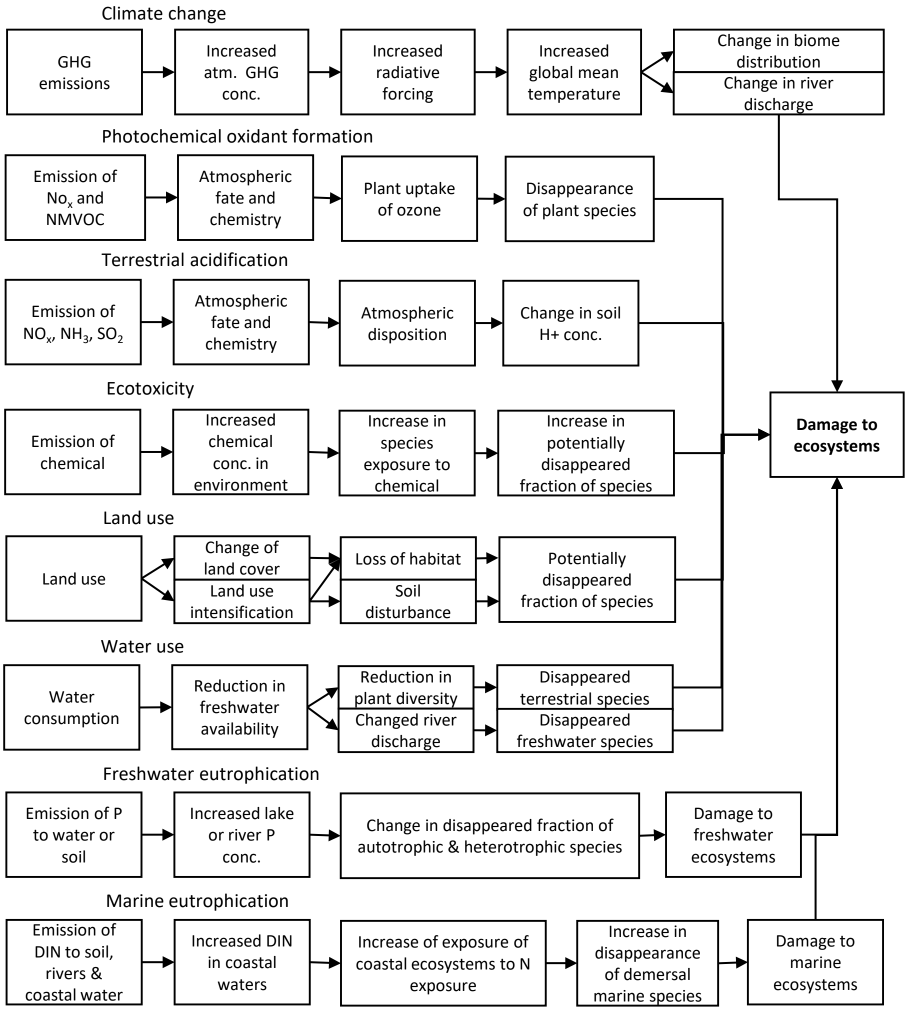

Supplementary Figure 15: **Cause-and-effect chain of midpoint indicators to ecosystem damage.** Figure based on ref.[74] GHG=Greenhouse gas emissions, NMVOC= Non-methane volatile organic compounds, DIN=Dissolved inorganic nitrogen, NH_3=ammonia, SO_2=sulfur dioxide, NO_x=nitrogen oxides.

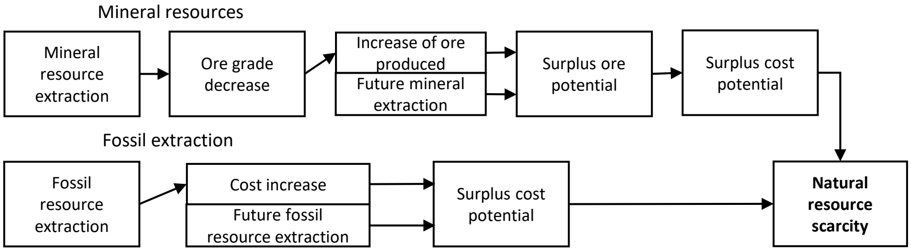

Supplementary Figure 16: **Cause-and-effect chain of midpoint indicators to natural resource scarcity.** Figure based on ref.[74] Natural resource scarcity is driven by mineral and fossil resource extraction.

Supplementary Discussion

Limitations and robustness of results

We likely underestimate the material stocks in fossil infrastructure, as we excluded oil refineries or internal combustion engine cars, as they could be refurbished into bio-refineries and electric cars. Stocks of steel and copper (1.34 Gt (0.84–2.26 Gt) and 10.03 Mt (5.94–18.33 Mt) are in the range of other estimates (0.5–2.0 Gt for steel and 9.3–38 Mt for copper).[31,32]

Further, we excluded biomass utilization in steel-making, which might reduce climate impacts of primary steel production, albeit at the trade-off with land and water use.

Recycling is one of several circular strategies that could be applied to obsolete infrastructures. Other strategies, such as reusing pipelines in district heating networks, retrofitting plants for carbon capture, or reusing power plant sites for grid storage infrastructure, could potentially avoid more costs and impacts or bring other advantages due to narrower cycles. Nevertheless, reusing will delay the available waste flow of materials, but will not reduce it, as even reused infrastructure will eventually be decommissioned. Future research can compare different circular strategies to find potential synergies or trade-offs.

We exclude dynamic implications of potentially accelerating the energy transitions by avoiding material supply bottlenecks for building renewable energy infrastructure (e.g. Al shortage). Especially in times of geopolitical tensions and the concurrent need to accelerate climate action, materials may not be available in the needed quantity and quality. Digesting fossil infrastructure may serve as a strategy to mitigate such supply shortages. The cumulative environmental benefit may thus be larger than the direct impact savings calculated in this study.

The general conclusions remain valid under pessimistic assumptions for scrap quality and slag composition and possible trade-offs with water use and ionizing radiation can be tackled by optimizing the electricity supply. Maintaining a high scrap quality is important, which can be achieved if waste flows from fossil infrastructure stay unmixed, reducing the need for dilution.

Leakage of Cr^{6+} from slags, which are produced in EAFs when chromium steel is present in the scrap, can undermine the benefits of steel recycling. Several EAF waste treatments exist that can reduce the leaching of toxic Cr^{6+} from slag[33–38] and dust,[39,40] but it is unclear how far these treatments are industrial practice. Adopting such slag and dust treatments is important to lower impact on human health.

We are assessing the total *potential* of materials that could be recycled from fossil infrastructure, not accounting for material stocks in infrastructure that might be inaccessible. This might be the case for subsea offshore infrastructure (pipelines, oil and gas rigs), however, only 2% of steel stocks and less than 0.01% of copper stocks respectively are located offshore (see Supplementary Fig. 1). Of these offshore stocks, 45% of steel and 98% of copper are stored in oil and gas wells, for which platform retrieval after decommissioning is common and feasible (e.g. in the North Sea,[41] although concrete jackets often remain). Offshore pipelines (around 1% of steel stocks) are often buried in the seafloor and not recovered.[42]

Other fossil infrastructure might be inaccessible because it is buried, such as oil and gas borehole equipment, pipelines, and underground mines. In closed boreholes, part of the oil and gas borehole equipment (steel casing) is left permanently in the ground to stabilize the borehole, whereas in open hole and barefoot wells, steel casing is not always required.[43] Under the assumption that all decommissioned wells need steel casing, we estimate the steel kept in ground as being 12% (3–23%) of gas and oil well infrastructure (see section below for details), representing around 6% (2–11%) of the total steel stock in fossil infrastructure. This fraction of the steel stock might be unavailable for recycling.

Additionally, steel in underground coal mines is used for structural support and might lead to ground instability if removed. Leaving steel in underground coal mines in place would reduce the available steel stock in fossil infrastructure for recycling by another 4% (see Supplementary Fig. 1).

In contrast, retrieval of onshore pipelines (18.6% and 0% of total global steel and copper stocks in fossil infrastructure) is technically feasible, although it might be uneconomical. Buried pipelines are allowed to be abandoned after use in some jurisdictions (e.g. in the US and the UK[44,45]). In cases where the economic value of the scrap does not compensate for the economic cost of retrieval, regulations would be needed that incentivize the removal of buried pipelines (or make removal mandatory) to ensure the availability of these stocks.

In total, the share of steel stocks that are inaccessible for recycling amounts to an estimated 11% (6–16%) and includes stocks in oil and gas well steel casings, underground mines, and offshore pipelines. Almost all copper stocks are likely accessible since they are not needed for structural purposes.

Permanent steel stock needed for well completion

In closed boreholes, steel casing remains in the hole for stabilization and is therefore not available for recycling. We estimate the steel stock in current global oil and gas wells that would remain underground if all wells included steel casing. We assume a casing used for intermediate holes (the longest part of the hole) with an outside diameter of 244.45 mm (medium size casing) and a steel mass of 79.62 kg m^{-1} (48.07–112.5 kg m^{-1}, varying with wall thickness).[46] For a well length of 3,000 m (1,000–5,000 m, see Supplementary Methods 7), this would yield a steel mass of 239 (48–563) ton per well unit, representing 12% (3–23%) of the steel mass of an average oil and gas rig respectively. Since oil and gas rigs account for 46% of steel stocks in fossil infrastructure (see Supplementary Fig. 1), the permanent steel casings represent around 6% (2–11)% of total steel stocks. We likely overestimate the steel mass needed for well completion because not all boreholes require casing over the whole length.

Carbon footprints of green hydrogen and methanol

We assess the cascading effect of greener production of renewable energy systems when using recycled steel from fossil infrastructure. As open-ground PV (ogPV) shows the largest climate impact reduction from using secondary steel and copper, we test the reduction potential in chemical production with open-ground PV in the case of Spain in the year 2025 following an SSP2-NPi scenario. The default case is a mix of primary steel, copper and aluminum in the open-ground PV, whereas Al is substituted with recycled steel from fossil infrastructure in the recycled case (ogPVr).

We test different types of electrolyzers (see Supplementary Fig. 17). The carbon footprint of hydrogen is reduced by 26% to 30% when supplying ogPVr electricity, achieving footprints below 1 kg $CO_{2,eq}$ kg^{-1}. This is lower than values found in the literature,[47] however, we use more up-to-date inventories for PV systems, more efficient single-Si instead of multi-Si cells, and lower impact mounting systems.

We test further processing of green hydrogen (using an PEM electrolyzer) into green methanol production by also testing the supply of ogPVr electricity in direct air capture CO_2 production. The combined effect yields a 6% reduction in the carbon footprint of green methanol compared to using default open-ground PV systems. Although this case study is not spatially or temporally representative of global green hydrogen production, it shows the potential of substituting for steel in renewable energy systems to reduce the footprints of energy-intensive chemicals.

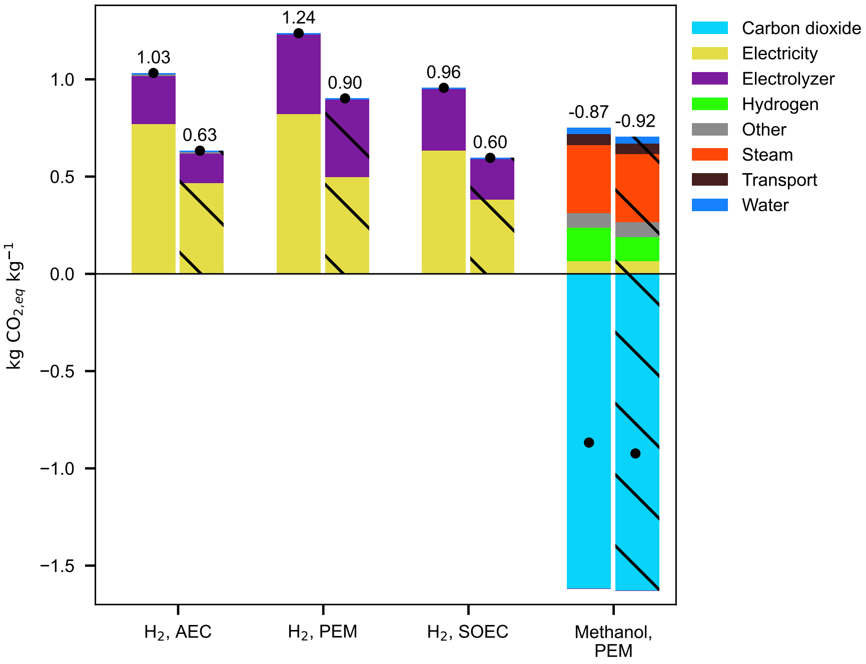

Supplementary Figure 17: **The carbon footprint of green hydrogen and green methanol production in Spain.** Business-as-usual (BAU) scenario reflects raw material production (hydrogen electrolysis and direct air capture of CO_2) electricity supply from PV with current Aluminum mounting systems (left bars). BAU scenario is compared to carbon footprint of green hydrogen and methanol production using recycled steel and copper in PV systems (right hatched bars). In both scenarios, the electricity source is open-ground PV following SSP2-NPi (Shared Socioeconomic Pathway 2 National Policies implemented) in the year 2025. SOEC=solid oxide electrolyzer, AEC=alkaline electrolyzer, PEM=proton exchange membrane electrolyzer.

Value of scrap in fossil infrastructure

For private fossil companies, the economic value of scrap could be an incentive for decommissioning infrastructure. Between February 2019 and February 2020, the ferrous scrap selling prices ranged from 257 to 479 USD_{2023} between regions.[48] For a back-of-the-envelope calculation, we assume a selling price of 350 USD_{2023} per ton and a potentially recyclable stock of 1.34 Gt steel scrap in current fossil infrastructure, yielding a total of 472 billion USD_{2023} worth of ferrous scrap in current global fossil infrastructure.

The copper scrap price ranged between 5,000 and 8,000 USD per ton in the second quarter of 2025.[49] To estimate the value of copper scrap, we assume of a selling price of 6,500 USD_{2023} per ton and a recyclable copper stock of 10.03 Mt. This yields a total copper value of 65 billion USD_{2023}. The total value of steel and copper scrap then amounts to 537 billion USD_{2023}.

Supplementary Tables

Supplementary Table 7: **Global annual production of materials shown in Figure 2 (main manuscript). PE=polyethylene, Cu=copper, Al=aluminum, Fe=iron, Zn=zinc, Ni=nickel, PVC=polyvinyl chloride, Cr=chromium, Co=cobalt, Si=silicon.**

Material	Total Production [Mt]	Year	Source
Concrete	3.00×10^{3}	2017	[55]
Sand	5.00×10^{4}	2022	[56]
Steel	1.90×10^{3}	2023	[57]
Wood	1.07×10^{3}*	2023	[58]
Act. bentonite	1.80×10^{1}	2021	[59]
Barite	8.50×10^{0}	2023	[60]
PE	1.05×10^{2}	2022	[61]
Cu	2.70×10^{1}	2023	[62]
Stone wool	1.00×10^{1}**	2021	[63]
Al	6.80×10^{1}	2021	[64]
Rubber	3.00×10^{1}	2022	[65]
Fe	1.30×10^{3}	2023	[57]
Lignite	6.39×10^{2}	2020	[66]
Zn	1.20×10^{1}	2023	[67]
Ni	3.60×10^{0}	2023	[68]
Epoxy resin	3.60×10^{0}	2022	[69]
PVC	5.10×10^{1}	2022	[61]
Cr	4.10×10^{1}	2021	[70]
Co	2.00×10^{-1}	2023	[71]
Si	3.80×10^{0}	2023	[72]

*1.92×10^{3} million m^3 assuming 555 kg m^{-3}, **forecast from 2016

Supplementary Table 8: **Steel and copper intensities of clean energy infrastructure. Intensity includes additional steel needed to replace aluminum in photovoltaic mounting systems. PEM=proton exchange membrane, SOEC=solid oxide electrolytic cell, AEC=alkaline electrolytic cell, MWp=megawatt peak capacity, km=kilometer.**

Technology	kg steel	kg copper	Per unit
photovoltaics, open-ground	5.51×10^{4}	3.40×10^{3}	MWp
photovoltaics, slanted-roof	2.98×10^{4}	1.01×10^{4}	MWp
wind energy, offshore	1.35×10^{5}	2.75×10^{3}	MWp
wind energy, onshore	1.52×10^{5}	9.83×10^{3}	MWp
electrolyzer, PEM	1.09×10^{4}	3.50×10^{2}	MWe
electrolyzer, SOEC	4.31×10^{4}	4.29×10^{2}	MWe
electrolyzer, AEC	3.81×10^{4}	6.17×10^{2}	MWe
transmission network	8.47×10^{2}	2.89×10^{3}	km

Supplementary Figures

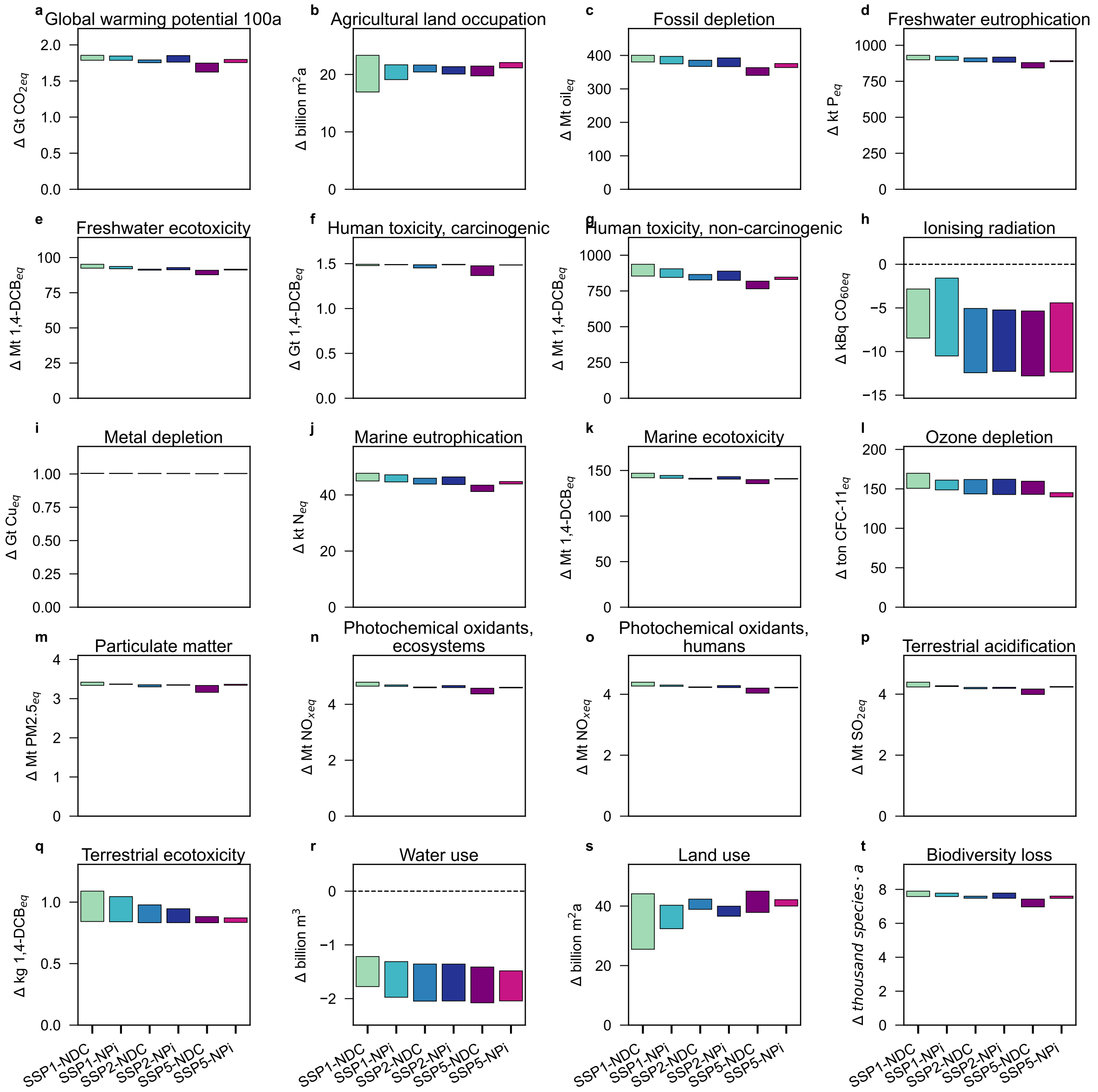

Supplementary Figure 18: **Total avoided environmental impacts through steel recycling (optimistic).** Bars show the minimum to maximum range of impact savings (midpoint indicators) of steel recycling compared to primary production for scenarios depending on the year of recycling (2025 to 2050). DCB=dichlorobenzene, CFC=chlorofluorocarbons, SSP=Shared socio-economic pathway, NPi= National policies implemented, NDC=National determined contributions. Gt=gigaton, Mt=megaton, kt=kiloton, kBq=kilobecquerel, DCB=Dichlorobenzene, PM2.5=Particulate matter of 2.5 micrometer equivalents, a=year.

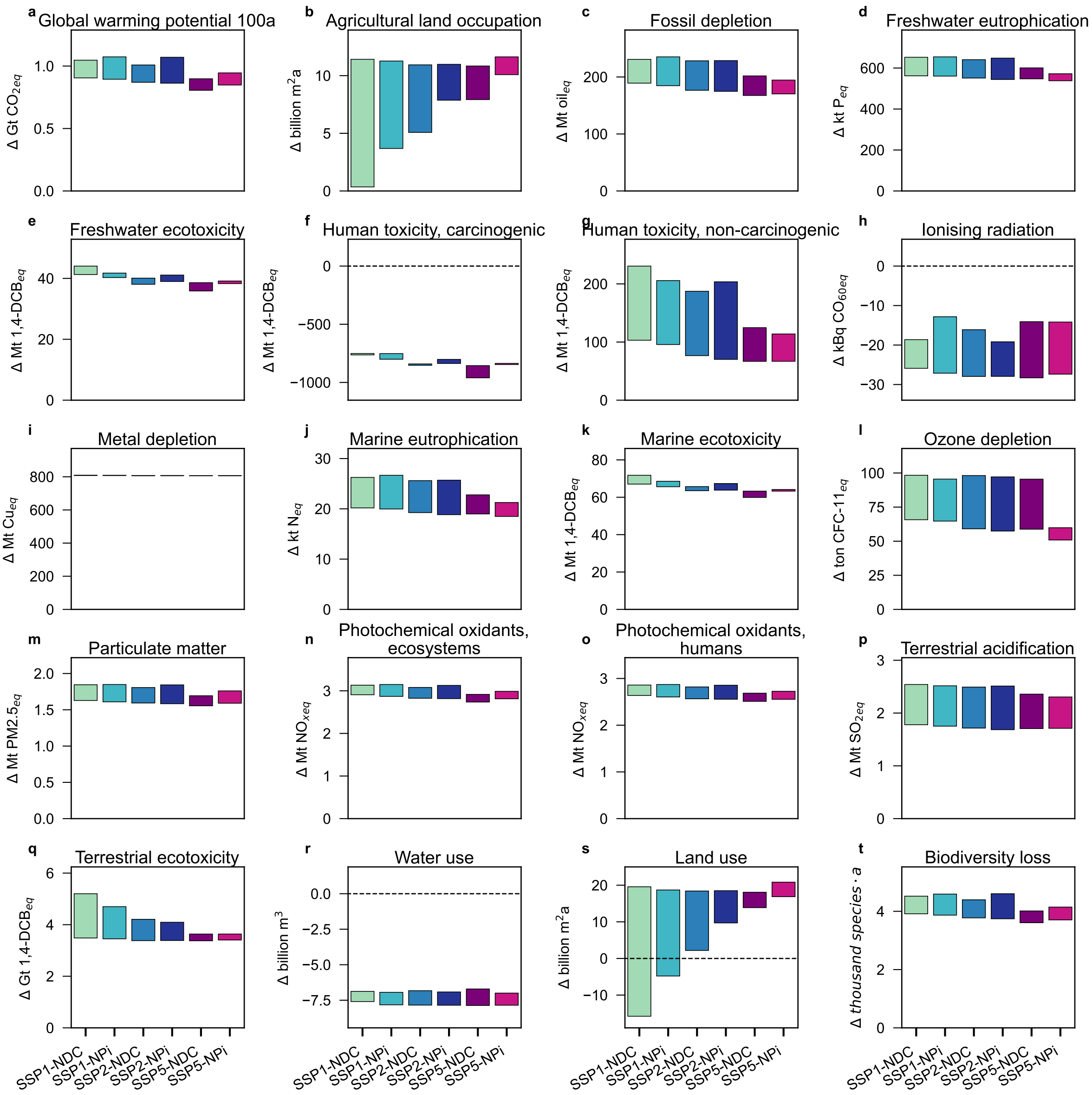

Supplementary Figure 19: **Total avoided environmental impacts through steel recycling (pessimistic).** Bars show the minimum to maximum range of impact savings (midpoint indicators) of steel recycling compared to primary production for scenarios depending on the year of recycling (2025 to 2050). DCB=dichlorobenzene, CFC=chlorofluorocarbons, SSP=Shared socioeconomic pathway, NPi= National policies implemented, NDC=National determined contributions. Gt=gigaton, Mt=megaton, kt=kiloton, kBq=kilobecquerel, DCB=Dichlorobenzene, PM2.5=Particulate matter of 2.5 micrometer equivalents, a=year.

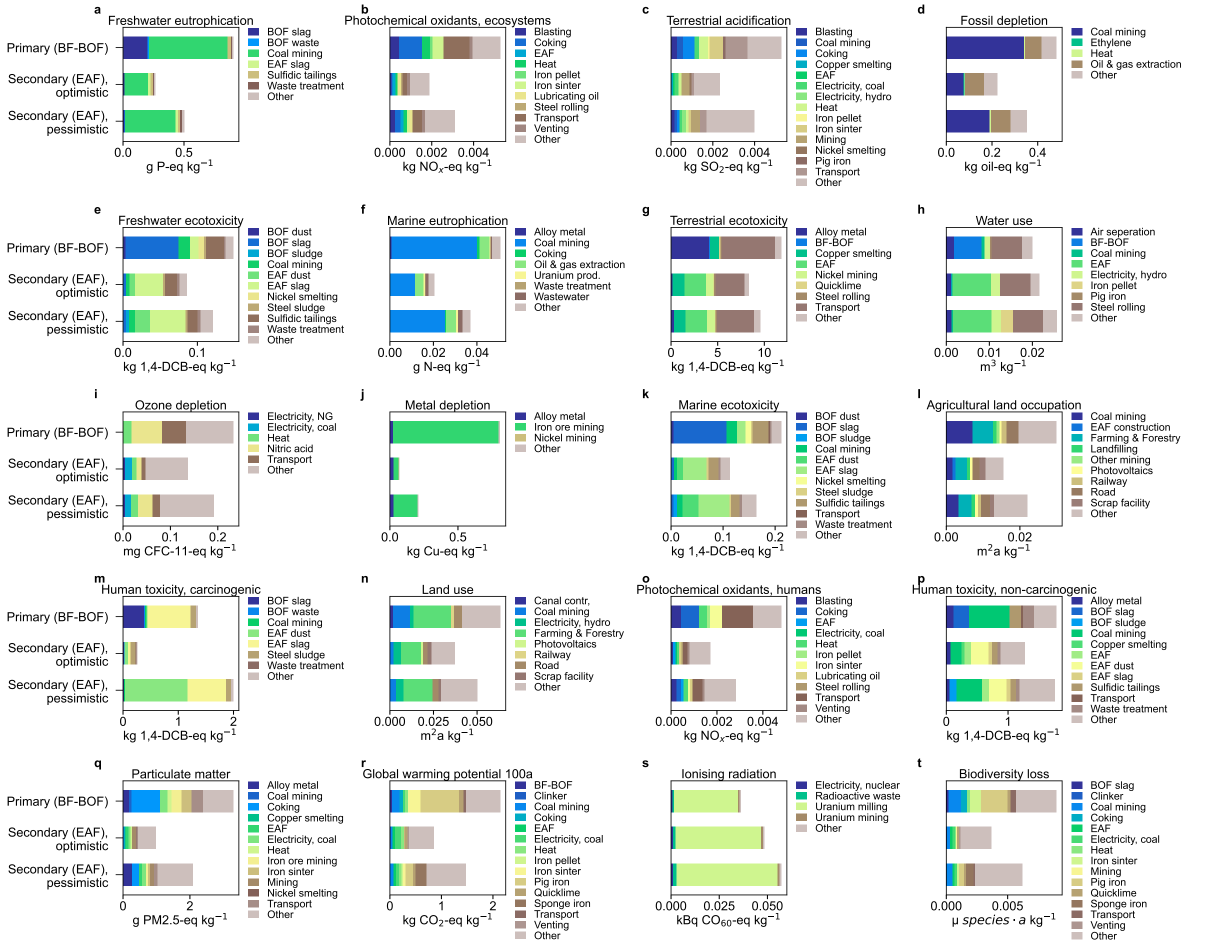

Supplementary Figure 20: **Process contributions to the environmental impacts of reinforcing steel.** Midpoint indicator impacts of 1 kg reinforcing steel are shown for the SSP2-NPi (Shared Socioeconomic Pathway 2 National Policies implemented) scenario in the year 2025. NG=natural gas, BF=blast furnace, BOF=basic oxygen furnace, EAF=electric arc furnace, PM=particulate matter, DCB=Dichlorobenzene, kBq=kilobecquerel, CFC=Chlorofluorocarbons.

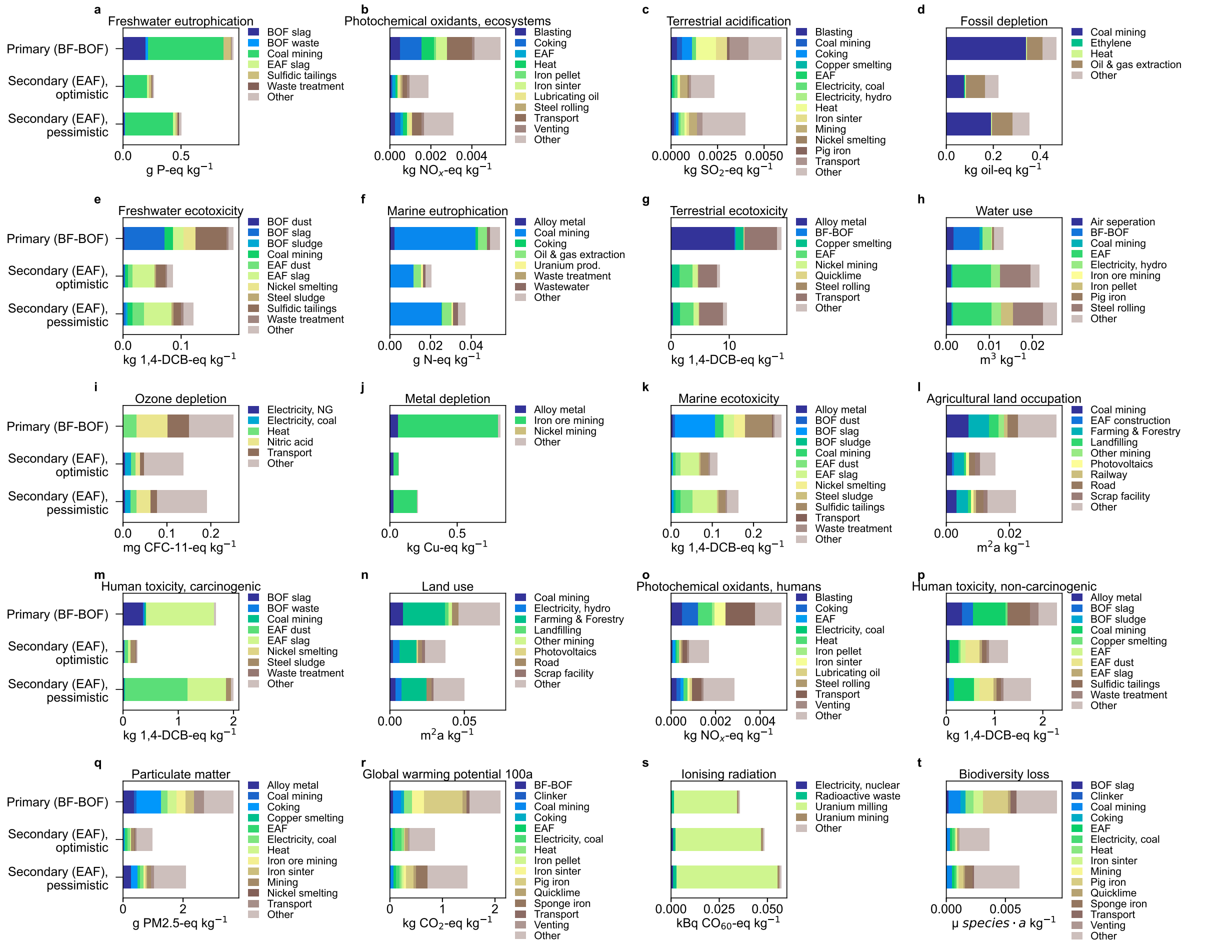

Supplementary Figure 21: **Process contributions to the environmental impacts of low-alloyed steel.** Midpoint indicator impacts of 1 kg low-alloyed steel are shown for the SSP2-NPi (Shared Socioeconomic Pathway 2 National Policies implemented) scenario in the year 2025. NG=natural gas, BF=blast furnace, BOF=basic oxygen furnace, EAF=electric arc furnace, PM=particulate matter, DCB=dichlorobenzene, kBq=kilobecquerel, kBq=kilobecquerel, CFC=chlorofluorocarbons.

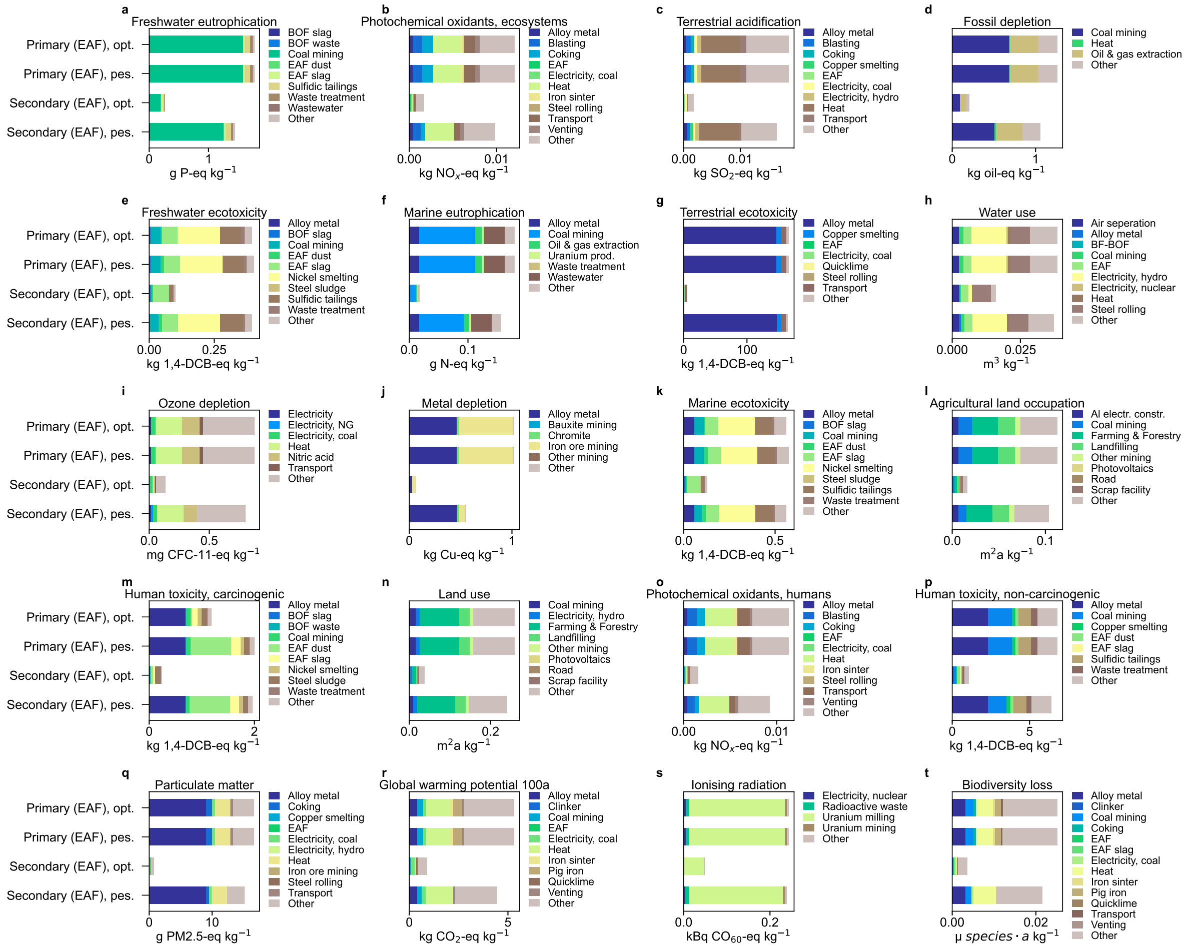

Supplementary Figure 22: **Process contributions to the environmental impacts of chromium steel.** Midpoint indicator impacts of 1 kg chromium steel are shown for the SSP2-NPi (Shared Socioeconomic Pathway 2 National Policies implemented) scenario in the year 2025. NG=natural gas, BF=blast furnace, BOF=basic oxygen furnace, EAF=electric arc furnace, PM=particulate matter, DCB=dichlorobenzene, kBq=kilobecquerel, CFC=chlorofluorocarbons.

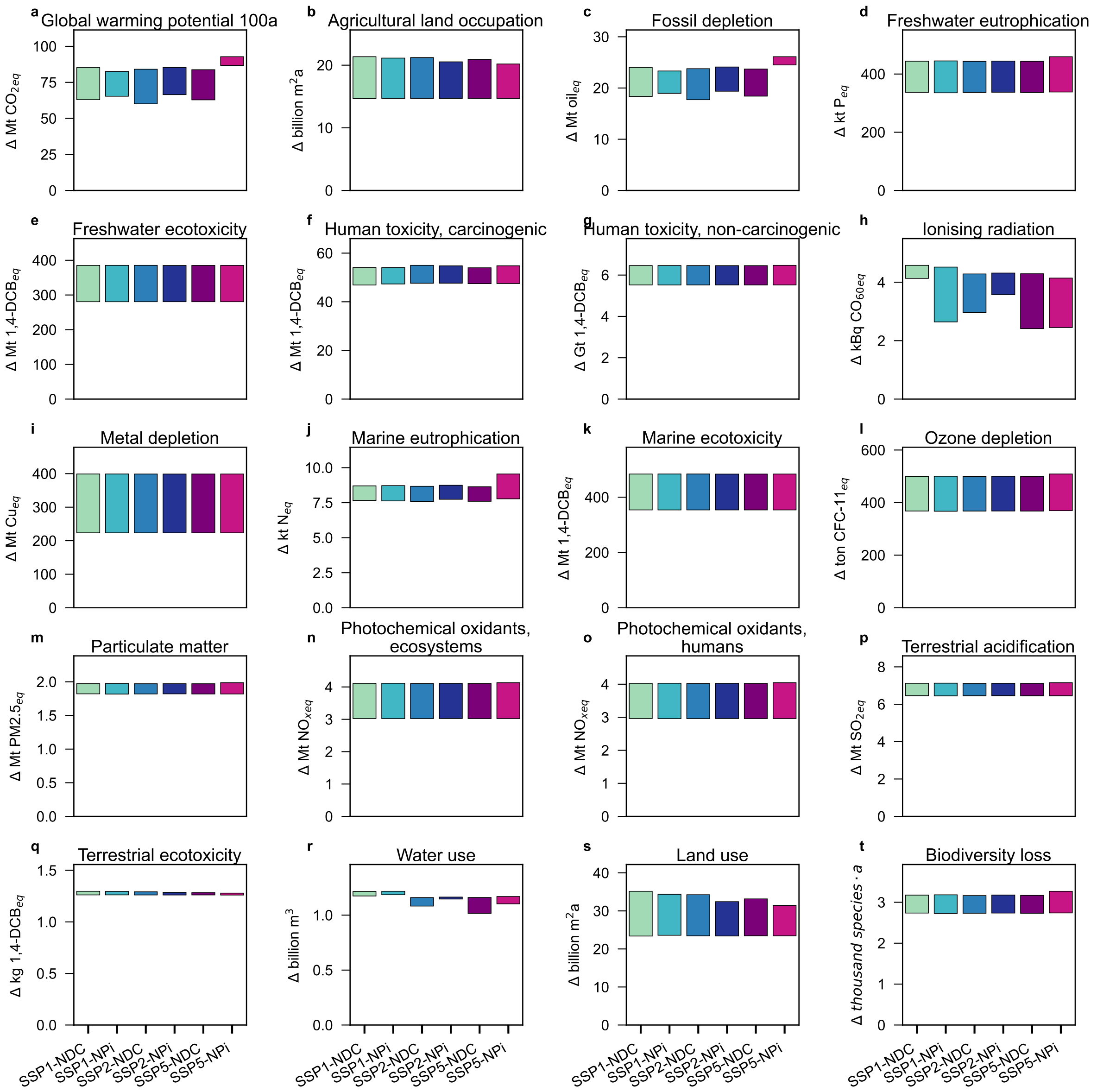

Supplementary Figure 23: **Total avoided environmental impacts through copper recycling (optimistic).** Bars show the minimum to maximum range of impact savings (midpoint indicators) of copper recycling compared to primary production for scenarios depending on the year of recycling (2025 to 2050). DCB=dichlorobenzene, CFC=chlorofluorocarbons, SSP=Shared socio-economic pathway, NPi= National policies implemented, NDC=National determined contributions. Gt=gigaton, Mt=megaton, kt=kiloton, kBq=kilobecquerel, DCB=Dichlorobenzene, PM2.5=Particulate matter of 2.5 micrometer equivalents, a=year.

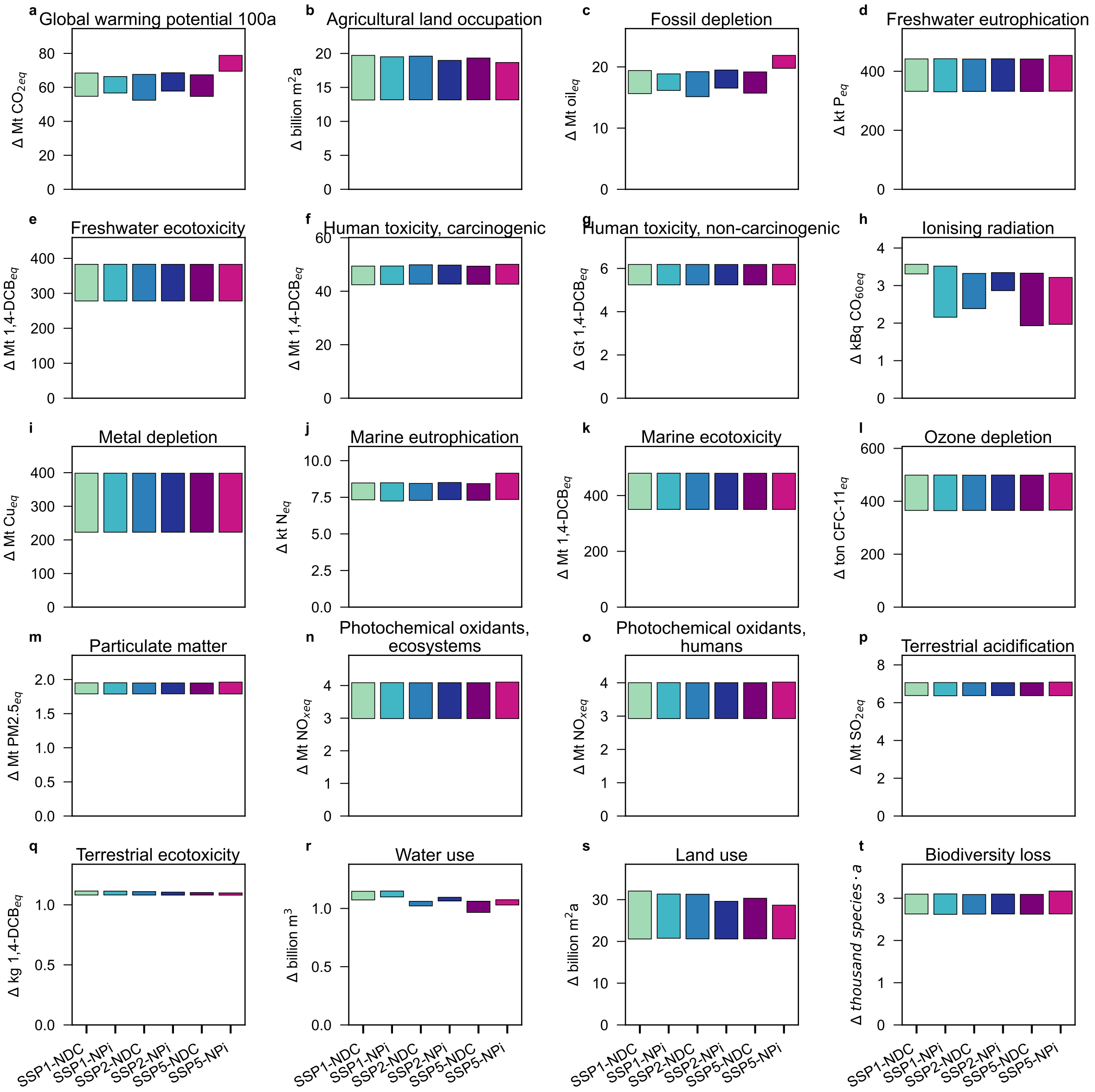

Supplementary Figure 24: **Total avoided environmental impacts through copper recycling (pessimistic).** Bars show the minimum to maximum range of impact savings (midpoint indicators) of copper recycling compared to primary production for scenarios depending on the year of recycling (2025 to 2050). DCB=dichlorobenzene, CFC=chlorofluorocarbons, SSP=Shared socioeconomic pathway, NPi= National policies implemented, NDC=National determined contributions. Gt=gigaton, Mt=megaton, kt=kiloton, kBq=kilobecquerel, DCB=Dichlorobenzene, PM2.5=Particulate matter of 2.5 micrometer equivalents, a=year.

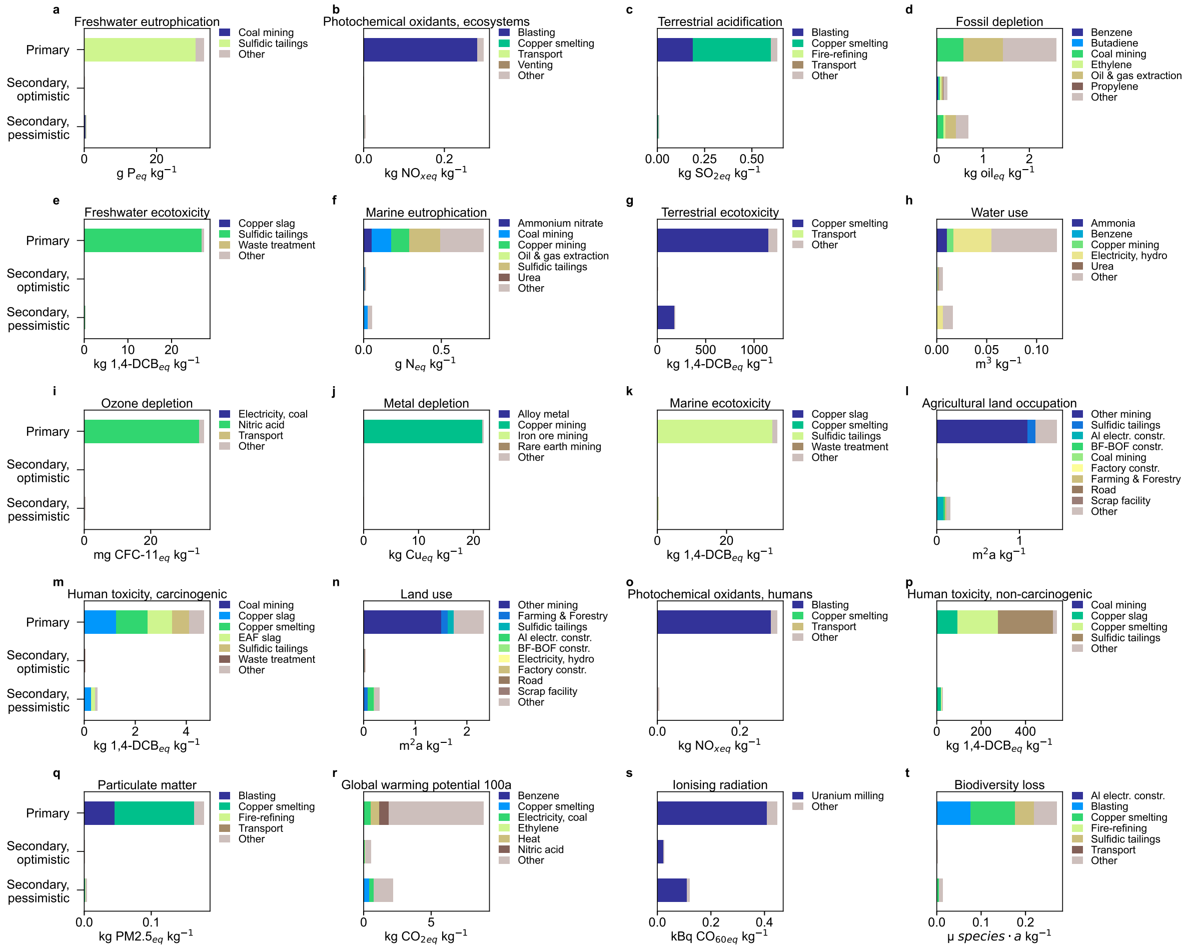

Supplementary Figure 25: **Process contributions to the environmental impacts of copper.** Midpoint indicator impacts of 1 kg copper (cathode) are shown for the SSP2-NPi (Shared Socioeconomic Pathway 2 National Policies implemented) scenario in the year 2025. NG=natural gas, BF=blast furnace, BOF=basic oxygen furnace, PM=particulate matter, DCB=dichlorobenzene, kBq=kilobecquerel, CFC=chlorofluorocarbons.

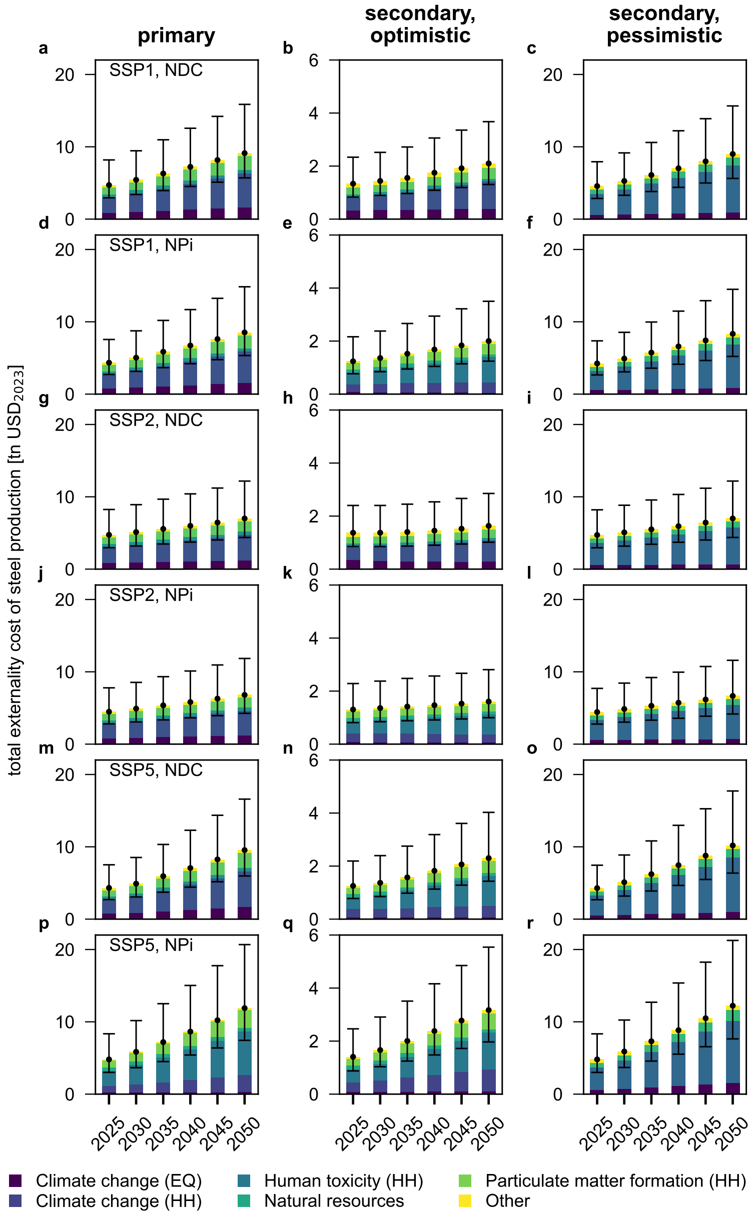

Supplementary Figure 26: **Externality costs of producing primary or secondary steel.** Bars show median total externality costs of steel production for scenario (rows) and production routes (columns) depending on the year of recycling. Error bars show the uncertainty of steel stock in fossil infrastructure (2.5th to 97.5th percentile of steel stock, measured with Monte Carlo simulation with n=500 runs of possible steel intensities). SSP=Shared socio-economic pathway, NPi= National policies implemented, NDC=National determined contributions, USD_{2023}=US Dollars in 2023 equivalent, HH=human health, EQ=Ecosystem quality.

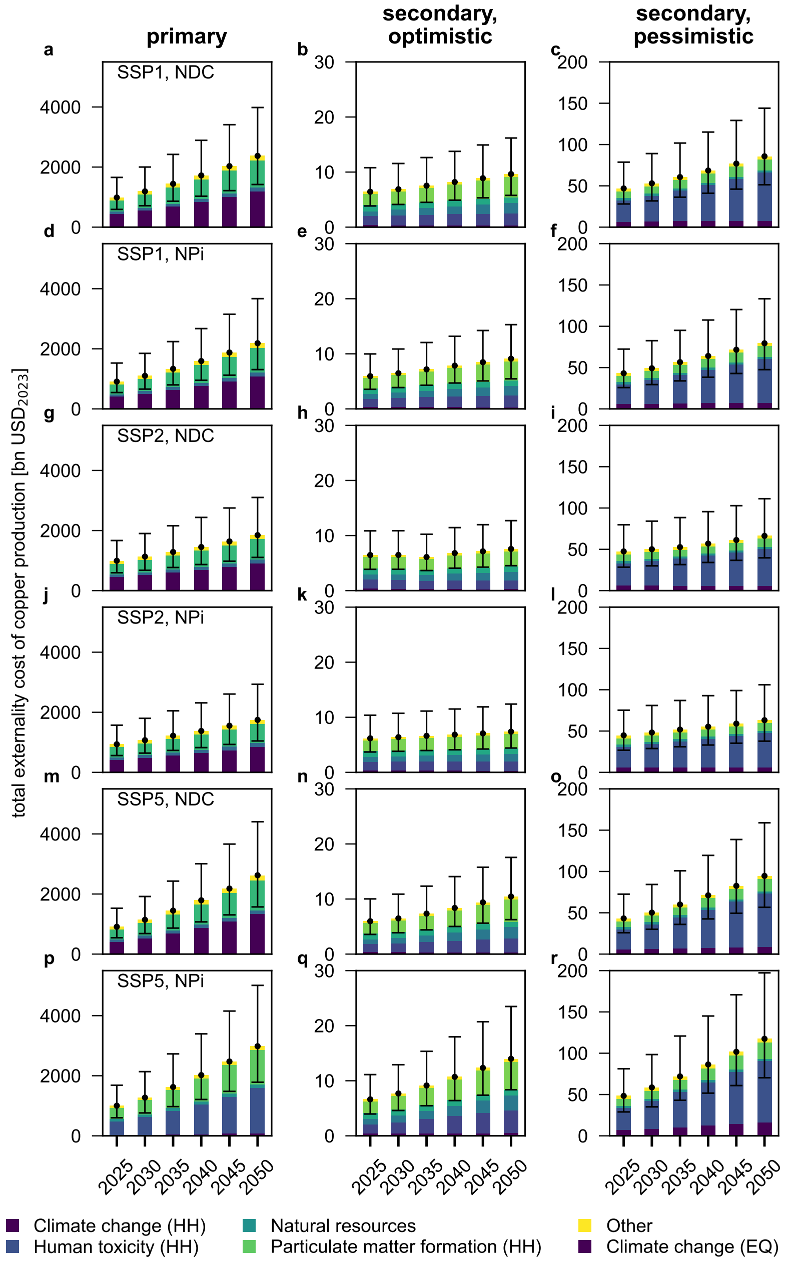

Supplementary Figure 27: **Externality costs of producing primary or secondary copper.** Bars show median total externality costs of copper production for scenario (rows) and production routes (columns) depending on the year of recycling. Error bars show the uncertainty of copper stock in fossil infrastructure (2.5th to 97.5th percentile of copper stock, measured with Monte Carlo simulation with n=500 runs of possible copper intensities).SSP=Shared socio-economic pathway, NPi= National policies implemented, NDC=National determined contributions, USD_{2023}=US Dollars in 2023 equivalent, HH=human health, EQ=Ecosystem quality.

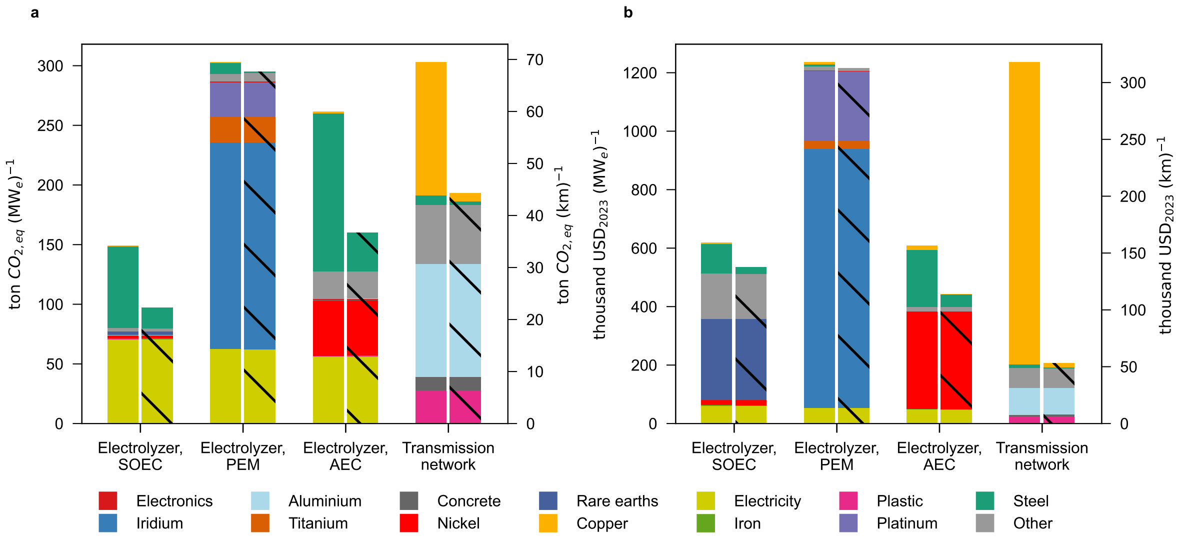

Supplementary Figure 28: **Environmental benefits of constructing hydrogen electrolyzers and electricity transmission infrastructure with recycled steel and copper**. **a**, Greenhouse gas emissions for hydrogen electrolyzers and transmission networks (medium voltage) constructed with primary steel and copper (left bars) compared to construction with recycled steel and copper (right hatched bars). Emissions for electrolyzers are shown on the left y-axis (per electrical capacity, MWe) and emissions for the transmission network on the right y-axis (per kilometer). **b**, Externality costs for electrolyzers and transmission network constructed with primary (left bars) and secondary (right hatched bars) steel and copper. SOEC=solid oxide electrolytic cell, PEM=proton exchange membrane, AEC=alkaline electrolytic cell, USD_{2023}=US Dollars in 2023 equivalents.

Supplementary References

[1] Sacchi, R. *Premise dashboard: dataset and visualisation.* Premise https://premisedash-6f5a0259c487.herokuapp.com (2025).

[2] International Copper Study Group. *World Copper Factbook 2024.* (International Copper Study Group, 2024). https://icsg.org/copper-factbook/.

[3] Sacchi, R. *et al.* Prospective environmental impact assement (premise): A streamlined approach to producing databases for prospective life cycle assessment using integrated assessment models. *Renew. Sustain. Energy Rev.* **160,** 112311 (2022). http://dx.doi.org/10.1016/j.rser.2022.112311.

[4] United Nations Framework Convention on Climate Change. *Paris Agreement.* (UNFCCC, Bonn, Germany, 2015). https://unfccc.int/process-and-meetings/the-paris-agreement/the-paris-agreement.

[5] Climate Action Tracker. *2100 warming projections: Emissions and expected warming based on pledges and current policies (November 2024).* Climate Analytics and NewClimate Institute. https://climateactiontracker.org/global/temperatures/ (2024)

[6] Remus, R., Aguado-Monsonet, M. A., Roudier, S., Delgado Sancho, L. *Best Available Techniques (BAT) Reference Document for Iron and Steel Production: Industrial Emissions Directive 2010/75/EU: Integrated Pollution Prevention and Control.* (Publications Office of the European Union, Luxembourg, 2013). https://doi.org/10.2791/97469.

[7] Wernet, G. *et al.* The ecoinvent database version 3 (part i): overview and methodology. *Int. J. Life Cycle Assess.* **21,** 1218–1230 (2016). www.doi.org/10.1007/s11367-016-1087-8.

[8] Lagos, G., Peters, D., Lima, M., Jara, J. J. Potential copper production through 2035 in Chile. *Miner. Econ.* **33,** 43–56 (2020). https://doi.org/10.1007/s13563-020-00227-2.

[9] Calvo, G., Mudd, G., Valero, A., Valero, A. Decreasing ore grades in global metallic mining: A theoretical issue or a global reality? *Resources* **5(4),** 36 (2016). https://doi.org/10.3390/resources5040036

[10] Davenport, W. G., King, M. J., Schlesinger, M. E. & Sole, K. C. Hydrometallurgical Copper Extraction: Introduction and Leaching. In *Extractive Metallurgy of Copper 5th Edition Ch. 15* pp. 281–322 (Elsevier, Oxford, 2011).

[11] Matsuoka, H., Mitsuhashi, K., Kawata, M., Tokoro, C. Derivation of flotation kinetic model for activated and depressed copper sulfide minerals. *Minerals* **11,** 1027 (2020) . https://doi.org/10.3390/min10111027.

[12] Davenport, W. G., King, M. J., Schlesinger, M. E. & Sole, K. C. Solvent Extraction. In *Extractive Metallurgy of Copper 5th Edition Ch. 16* pp. 323–347 (Elsevier, Oxford, 2011).

[13] Davenport, W. G., King, M. J., Schlesinger, M. E. & Sole, K. C. Electrowinning. In *Extractive Metallurgy of Copper 5th Edition Ch. 17* pp. 349–372 (Elsevier, Oxford, 2011).

[14] Davenport, W. G., King, M. J., Schlesinger, M. E. & Sole, K. C. Fire Refining (S and O Removal) and Anode Casting. In *Extractive Metallurgy of Copper 5th Edition Ch. 13* pp. 237–249 (Elsevier, Oxford, 2011).

[15] Kalliomaki, T., Aji, A. T., Rintala, L., Aromaa, J., Lundstrom, M. Models for viscosity and density of copper electrorefining electrolytes. *Physicochem. Probl. Miner. Process.* **53,** 1023–1037 (2017). https://doi.org/10.5277/ppmp170227.

[16] Davenport, W. G., King, M. J., Schlesinger, M. E. & Sole, K. C. Electrolytic Refining. In *Extractive Metallurgy of Copper 5th Edition Ch. 14* pp. 251–280 (Elsevier, Oxford, 2011).

[17] Eastwood, K. L. & Whebell, G. W. Developments in permanent stainless steel cathodes within the copper industry. *Xstrata Technology Technical Paper* (2006).

[18] Davenport, W. G., King, M. J., Schlesinger, M. E. & Sole, K. C. Converting of Copper Matte. In *Extractive Metallurgy of Copper 5th Edition Ch. 8* pp. 127–153 (Elsevier, Oxford, 2011).

[19] Classen, M. *et al. Life Cycle Inventories of Metals.* Final Report, ecoinvent Data v2.1, No. 10 (ecoinvent Centre, Dübendorf, Switzerland, 2009).

[20] Welling, R. *et al.* Chromium VI and stomach cancer: a meta-analysis of the current epidemiological evidence. *Occup. Environ. Med.* **72,** 151–159 (2014).

[21] Prasad, S. *et al.* Chromium contamination and effect on environmental health and its remediation: A sustainable approaches. *J. Environ. Manag.* **285,** 112174 (2021).

[22] Proctor, D. M. *et al.* Physical and chemical characteristics of blast furnace, basic oxygen furnace, and electric arc furnace steel industry slags. *Environ. Sci. Technol.* **34,** 1576–1582 (2000).

[23] Omran, M. & Fabritius, T. Effect of steelmaking dust characteristics on suitable recycling process determining: Ferrochrome converter (CRC) and electric arc furnace (EAF) dusts. *Powder Technol.* **308,** 47–60 (2017).

[24] Müller, A. *et al.* A comparative life cycle assessment of silicon PV modules: Impact of module design, manufacturing location and inventory. *Sol. Energy Mater. Sol. Cells* **230,** 111277 (2021). https://doi.org/10.1016/j.solmat.2021.111277.

[25] TransitionZero. *Global Steel Cost Tracker.* TransitionZero https://www.transitionzero.org/products/global-steel-cost-tracker (2022)

[26] Aguirregabiria, V. & Luengo, A. A microeconometric dynamic structural model of copper mining decisions. *Unpublished manuscript.* Department of Economics, University of Toront. (2016). http://aguirregabiria.net/wpapers/copper_mining.pdf.

[27] International Renewable Energy Agency. *Renewable Power Generation Costs in 2023*. https://www.irena.org/-/media/Files/IRENA/Agency/Publication/2024/Sep/IRENA_Renewable_power_generation_costs_in_2023.pdf (International Renewable Energy Agency, Abu Dhabi, 2024).

[28] Global Energy Monitor. *Global Coal Mine Tracker, July 2022 release*. https://globalenergymonitor.org/projects/global-coal-mine-tracker/ (2022).

[29] Parish, E. S. *et al.* Comparing scales of environmental effects from gasoline and ethanol production. *Environ. Manag.* **51,** 307–338 (2013).

[30] Huijbregts, M. *et al.* Recipe2016: a harmonised life cycle impact assessment method at midpoint and endpoint level. *Int. J. Life Cycle Assess.* **22,** 138–147 (2017). www.doi.org/10.1007/s11367-016-1246-y.

[31] Le Boulzec, H. *et al.* Dynamic modeling of global fossil fuel infrastructure and materials needs: Overcoming a lack of available data. *Appl. Energy* **326,** 119871 (2022).

[32] Deetman, S., de Boer, H. S., Van Engelenburg, M., van der Voet, E. & van Vuuren, D. P. Projected material requirements for the global electricity infrastructure–generation, transmission and storage. *Resour. Conserv. Recycl.* **164,** 105200 (2021).

[33] Tae, S. & Morita, K. Immobilization of Cr (VI) in stainless steel slag and Cd, As, and Pb in wastewater using blast furnace slag via a hydrothermal treatment. *Met. Mater. Int.* **23,** 576–581 (2017).

[34] Lin, Y., Yan, B., Fabritius, T. & Shu, Q. Immobilization of chromium in stainless steel slag using low zinc electric arc furnace dusts. *Metall. Mater. Trans. B* **51,** 763–775 (2020). http://dx.doi.org/10.1007/s11663-020-01777-0.

[35] Strandkvist, I. *et al.* Minimizing chromium leaching from low-alloy electric arc furnace (EAF) slag by adjusting the basicity and cooling rate to control brownmillerite formation. *Appl. Sci.* **10,** 35 (2019). http://dx.doi.org/10.3390/app10010035.

[36] Spanka, M., Mansfeldt, T. & Bialucha, R. Influence of natural and accelerated carbonation of steel slags on their leaching behavior. *Steel Res. Int.* **87,** 798–810 (2016). http://dx.doi.org/10.1002/srin.201500370.

[37] Mombelli, D., Gruttadauria, A. & Mapelli, C. The influence of slag tapping method on the efficiency of stabilization treatment of electric arc furnace carbon steel slag (EAF-C). *Minerals* **9,** 706 (2019). http://dx.doi.org/10.3390/min9110706.

[38] Cao, S., Sohn, I. & Wang, Z. Selective stabilization of chromium and sustainable treatment of stainless steel slags. *J. Environ. Chem. Eng.* **12,** 113516 (2024). http://dx.doi.org/10.1016/j.jece.2024.113516.

[39] Wang, J. *et al.* Pyrometallurgical recovery of zinc and valuable metals from electric arc furnace dust–a review. *J. Clean. Prod.* **298,** 126788 (2021).

[40] Xu, J. *et al.* Valuable recovery technology and resource utilization of chromium-containing metallurgical dust and slag: a review. *Metals* **13,** 1768 (2023).

[41] OSPAR Commission. *OSPAR Decision 98/3 on the Disposal of Disused Offshore Installations (as amended by OSPAR Decision 2024/01): Consolidated Text*. https://www.ospar.org/documents?v=57705 (OSPAR Commission, London, 2024).

[42] Efthymiou, M. Decommissioning of Offshore Oil and Gas Installations. In *Encyclopedia of Ocean Engineering* pp. 275–287 (Springer, Singapore, 2022). https://doi.org/10.1007/978-981-10-6946-8_230.

[43] Crumpton, H. Well Construction and Completion Design. In *Well Control for Completions and Interventions* pp. 65–85 (Elsevier, Oxford, 2018). https://doi.org/10.1016/B978-0-08-100196-7.00002-6.

[44] U.S. Department of Transportation, Pipeline and Hazardous Materials Safety Administration. *49 CFR §192.727 – Abandonment or deactivation of facilities*. https://www.law.cornell.edu/cfr/text/49/192.727 (Pipeline and Hazardous Materials Safety Administration, Washington, DC, 2024).

[45] UK Onshore Pipeline Operators' Association (UKOPA). *Good Practice Guide: Pipeline Decommissioning and Abandonment (GPG/038)*. https://ukopa.co.uk/wp-content/uploads/2025/04/GPG038-Decommissioning-and-Abandonment-V1.0-apprpoved-Oct-2023.pdf (UK Onshore Pipeline Operators' Association (UKOPA), Ambergate, Derbyshire, 2023).

[46] American Petroleum Institute. *API Spec 5CT – Specification for Casing and Tubing*. https://www.octalsteel.com/pdf/api-spec-5ct-specification-for-casing-and-tubing.pdf (American Petroleum Institute, Washington, DC, 2011).

[47] Weidner, T., Tulus, V. & Guillén-Gosálbez, G. Environmental sustainability assessment of large-scale hydrogen production using prospective life cycle analysis. *Int. J. Hydrog. Energy* **48,** 8310–8327 (2023). http://dx.doi.org/10.1016/j.ijhydene.2022.11.044.

[48] Intratec. *Ferrous Scrap Prices | Current and Forecast*. https://www.intratec.us/chemical-markets/ferrous-scrap-price (2025; accessed 28 Feb 2025).

[49] IMARC Group. *Copper Scrap Prices, Trend, Chart, Demand, Market Analysis, News, Historical and Forecast Data Report 2025 Edition*. https://www.imarcgroup.com/copper-scrap-pricing-report (IMARC Group, Sheridan, WY, 2025).

[50] Chepushtanova, T., Yessirkegenov, M., Mamyrbayeva, K., Akcil, A., Gaipov, T. Extraction of copper from pregnant leach solution (PLS) and reduction of crud formation. *Miner. Process. Extr. Metall. Rev.* **46(4),** 479–491 (2024), 10.1080/08827508.2024.2340545.

[51] Ochromowicz, K., Chmielewski, T. Solvent extraction of copper (II) from concentrated leach liquors. *Physicochem. Probl. Miner. Process.* **49(1),** 357–367 (2013).

[52] Hernández, J., Tapia, J. Direct copper recovery from pregnant leaching solutions (PLS), using a custom electrolytic cell, based on reactive electrodialysis (RED). *Miner. Process. Extr. Metall.* **132(2),** 110–116 (2023).

[53] Zeng, X. *et al.* Comparing the costs and benefits of virgin and urban mining. *J. Manag. Sci. Eng.* **7,** 98–106 (2022). http://dx.doi.org/10.1016/j.jmse.2021.05.002.

[54] Boulamanti, A., Moya, J. A. & Production costs of the non-ferrous metals in the EU and other countries: Copper and zinc. *Res. Policy* **49,** 112–118 (2016). https://doi.org/10.1016/j.resourpol.2016.04.011.

[55] Monteiro, P. J. M., Miller, S. A. & Horvath, A. Towards sustainable concrete. *Nat. Mater.* **16,** 698–699 (2017).

[56] Vander Velpen, A. *et al. Sand and Sustainability: 10 Strategic Recommendations to Avert a Crisis*. (United Nations Environment Programme, Nairobi, Kenya, 2022).

[57] U.S. Geological Survey. *Mineral Commodity Summaries 2024: Iron and Steel*. https://pubs.usgs.gov/periodicals/mcs2024/mcs2024-iron-steel.pdf (U.S. Geological Survey, Reston, VA, 2024).

[58] Food and Agriculture Organization of the United Nations. *Global Forest Products Facts and Figures 2023*. https://doi.org/10.4060/cd3650en (Food and Agriculture Organization of the United Nations, Rome, 2024).

[59] U.S. Geological Survey. *Mineral Commodity Summaries 2022: Clays*. https://pubs.usgs.gov/periodicals/mcs2022/mcs2022-clays.pdf (U.S. Geological Survey, Reston, VA, 2022).

[60] U.S. Geological Survey. *Mineral Commodity Summaries 2024: Barite*. https://pubs.usgs.gov/periodicals/mcs2024/mcs2024-barite.pdf (U.S. Geological Survey, Reston, VA, 2024).

[61] PlasticsEurope. *Plastics – The Fast Facts 2023*. https://plasticseurope.org/de/wp-content/uploads/sites/3/2023/11/PlasticsthefastFacts2023_printing.pdf (PlasticsEurope, Brussels, Belgium, 2023).

[62] U.S. Geological Survey. *Mineral Commodity Summaries 2024: Copper*. https://pubs.usgs.gov/periodicals/mcs2024/mcs2024-copper.pdf (U.S. Geological Survey, Reston, VA, 2024).

[63] Statista. *Market Volume of the Insulation Material Market Worldwide in 2016, with a Forecast for 2021, by Material*. https://www.statista.com/statistics/911536/insulation-material-market-volume-worldwide-by-material/ (2016; accessed 29 Dec 2024).

[64] U.S. Geological Survey. *Mineral Commodity Summaries 2022: Aluminum*. https://pubs.usgs.gov/periodicals/mcs2022/mcs2022-aluminum.pdf (U.S. Geological Survey, Reston, VA, 2022).

[65] *Consumption of natural and synthetic rubber worldwide from 1990 to H1 2024*. (Statista, 2024). Last accessed December 29th 2024 at https://www.statista.com/statistics/911536/insulation-material-market-volume-worldwide-by-material/.

[66] International Energy Agency. *Coal Information*. https://www.iea.org/reports/coal-information-overview/production (International Energy Agency, Paris, 2021).

[67] U.S. Geological Survey. *Mineral Commodity Summaries 2024: Zinc*. https://pubs.usgs.gov/periodicals/mcs2024/mcs2024-zinc.pdf (U.S. Geological Survey, Reston, VA, 2024).

[68] U.S. Geological Survey. *Mineral Commodity Summaries 2024: Nickel*. https://pubs.usgs.gov/periodicals/mcs2024/mcs2024-nickel.pdf (U.S. Geological Survey, Reston, VA, 2024).

[69] ChemAnalyst. *Epoxy Resin Market Size, Growth, and Forecast, 2030*. https://www.chemanalyst.com/industry-report/epoxy-resin-market-597 (2024; accessed 25 Mar 2025).

[70] U.S. Geological Survey. *Mineral Commodity Summaries 2022: Chromium*. https://pubs.usgs.gov/periodicals/mcs2022/mcs2022-chromium.pdf (U.S. Geological Survey, Reston, VA, 2022).

[71] Energy Institute. *Statistical Review of World Energy (2024)*. https://www.energyinst.org/statistical-review (Energy Institute, London, 2024).

[72] U.S. Geological Survey. *Mineral Commodity Summaries 2024: Silicon*. https://pubs.usgs.gov/periodicals/mcs2024/mcs2024-silicon.pdf (U.S. Geological Survey, Reston, VA, 2024).

[73] Byers, E. *et al. AR6 Scenarios Database*. Zenodo https://doi.org/10.5281/zenodo.5886911 (2022).

[74] Huijbregts, M.A.J. *et al. ReCiPe 2016 v1.1: A Harmonized Life Cycle Impact Assessment Method at Midpoint and Endpoint Level. Report I: Characterization*. https://www.rivm.nl/documenten/recipe2016v11 (National Institute for Public Health and the Environment (RIVM), Bilthoven, The Netherlands, 2017).
